# Chemical Memory Reactions Induced Bursting Dynamics in Gene Expression

**DOI:** 10.1371/journal.pone.0052029

**Published:** 2013-01-21

**Authors:** Tianhai Tian

**Affiliations:** School of Mathematical Science, Monash University, Melbourne, Victoria, Australia; Semmelweis University, Hungary

## Abstract

Memory is a ubiquitous phenomenon in biological systems in which the present system state is not entirely determined by the current conditions but also depends on the time evolutionary path of the system. Specifically, many memorial phenomena are characterized by chemical memory reactions that may fire under particular system conditions. These conditional chemical reactions contradict to the extant stochastic approaches for modeling chemical kinetics and have increasingly posed significant challenges to mathematical modeling and computer simulation. To tackle the challenge, I proposed a novel theory consisting of the memory chemical master equations and memory stochastic simulation algorithm. A stochastic model for single-gene expression was proposed to illustrate the key function of memory reactions in inducing bursting dynamics of gene expression that has been observed in experiments recently. The importance of memory reactions has been further validated by the stochastic model of the p53-MDM2 core module. Simulations showed that memory reactions is a major mechanism for realizing both sustained oscillations of p53 protein numbers in single cells and damped oscillations over a population of cells. These successful applications of the memory modeling framework suggested that this innovative theory is an effective and powerful tool to study memory process and conditional chemical reactions in a wide range of complex biological systems.

## Introduction

Recent experimental studies in single cells have shown that gene expression is governed by stochastic process [1], [2], [3]. Randomness in transcription and translation leads to cell-to-cell variations at both message RNA (mRNA) and protein levels. Following the observation of translational bursts [4], [5], single-cell studies demonstrated that gene transcription also occurred in bursts of multiple transcripts separated by relatively long periods of transcriptional inactivity [6], [7], [8]. The length of inactivity windows varies widely for different genes, from a few minutes in prokaryotic cells to approximately a few hours in eukaryotic cells [6], [9]. In addition, varying numbers of gene expression pulses were observed in identical cells that were exposed to the same experimental conditions. The plausible mechanisms underlying transcriptional bursts include stochastic events of chromatin remodeling, existence of pre-initiation complexes, and competition of transcription factories [10], [11], [12]. However, such stochastic expression events also have certain deterministic properties. For example, the length and amplitude of these bursts are fairly constant in experiments using different extra-cellular stimulations [7]. Although the evidence of transcriptional bursting continues to accumulate, the mechanisms for inducing the bursts are still not fully understood.

There are a variety of modeling approaches to describe the bursting dynamics of gene expression. Early research works used the Poisson process to generate burst events in transcription and translation instantly [13], [14]. Similar approaches, which are called the random telegraph model, have also been used to provide insightful information regarding the importance of promoter activity [6], [14], [15], [16], [17], [18]. Another stochastic model assumed that genes switched slowly between active and inactive states and mRNA synthesis occurs only during the active stage [1]. In addition, a more general model was designed to study the effect of process that can give rise to “gestation” and “senescence” period of mRNA birth and decay [19]. Recently, a stochastic model was developed to study the stochastic bursting including agent-like actions in which the slow bursting of the GAL1 gene was explained by a production of an agent-like inhibitor after the induction process causes a refractory state in the promoter [20]. However, recent experimental studies of mRNA distributions have provided strong evidence for transcriptional noise beyond what can be described by a simple Poisson process [21]. Therefore more realistic stochastic models are indispensable to investigate the dynamics of burst events accurately.

The stochastic simulation algorithm (SSA) represents an essentially exact procedure for numerically simulating the time evolution of a well-stirred reaction system [22]. This simulation method has been extended to study chemical systems with time-dependent and non-Markov processes [23]. To investigate the function of noise in slow reactions and multiple step chemical reactions, the delay stochastic simulation algorithm (delay-SSA) was proposed to incorporate time delay, intrinsic noise, and discreteness associated with chemical kinetic systems into a single framework [24], [25]. The delay-SSA was extended to describe chemical events that have multiple delays and that the time delays may be distributed (i.e. random variables) [26]. In recent years, this effective modelling framework has been widely used to describe the complex dynamics of biological systems, including genetic regulatory networks and cell signalling pathways [27], [28], [29], [30], [31]. In addition, effective numerical methods have been proposed to accelerate stochastic simulations for biological systems with time delay [32]. When using time delay to represent multiple step reactions, it was assumed that the intermediate products of small step reactions did not involve in any other reactions of the system. However, if the intermediate products involve in certain specific chemical reactions and play important roles during the delay time period, we regard these chemical reactions have certain memory property. Thus more sophisticated modeling schemes are needed to describe the chemical reactions having complex properties.

Memory is a ubiquitous phenomenon in biological systems [33], [34], [35]. In psychology, memory is an organism's ability to store, retain, and recall information and experiences. In addition to the conventional function of the brain, memory has been used in systems biology recently to investigate the ability of small systems to store information. For example, cellular memory has been used to describe the ability of biological systems to maintain sustained response to a transient stimulus as well as two or more discrete stable states [36], [37], [38]. In addition, molecular memory has been proposed to describe chemical events consisting of several small step reactions [19]. The common characteristics of the memory phenomena is that the present system state is not entirely determined by current conditions but also depends on the past history of the system [33]. Thus the firing of certain chemical reactions in a memory system is conditional to the past system states and past chemical events. These conditional chemical reactions defy the fundamental assumption of chemical kinetics and have not been addressed before by using mathematical modeling approaches. To tackle the challenge, this work develops a novel modeling and simulation framework to describe biological systems with memory. Using the p53-MDM2 core circuit as the model system, we illustrate the roles of memory reactions in generating bursting events in gene expression.

## Methods

### Chemical memory reaction

This work first proposed a novel theory to model biological systems with chemical memory reactions. Chemical reactions in the system are classified into (non-memory) reactions and memory reactions; and each category contains elementary reactions and delayed reactions. Defined as chemical reaction firing in the path of a molecular memory event, memory reaction may occur during particular time-periods and/or under specific system conditions. An example of the memory events is the refractory time period during which an organ or cell is incapable of repeating a particular action. In gene expression, one of the refractory states is the chromatin epigenetic process, such as silencing by DNA methylation and structural changes in chromatin [39], [40]. Since silencing molecules are recruited by an autocatalytic mechanism, this can lead to a long periods of reactivation, as exemplified by the ON/OFF switching in the epigenetic silencing by Sir3 [41] and a refractory period of transcriptional inactivation close to 3 h in mammalians [42].

During the time period of transcriptional activation, both the transcriptional factor (TF) and RNA polymerase (RNAP) can bind to the corresponding promoter site, which has been modeled by the following elementary reactions
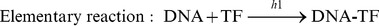
(1)

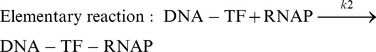
(2)These reactions have been widely used in the stochastic models for studying gene expression. However, experimental observations suggested that, during the refractory period, the transcriptional activators could gain access to silenced chromatin but that RNAP and TATA-binding protein (TBP) are excluded [43], [44]. Therefore reaction (Eq. 1) may fire but reaction (Eq. 2) be unable to fire during the silencing time period. A new reaction is needed to realize the event in the refractory period. Such reaction is defined as memory reaction in this work. The time period during which memory reactions may fire is termed as the memory time period. The length of a memory time period may be either a constant or a random variable with an associated probability distribution. The probability distribution used in this work is either the exponential distribution or Gaussian distribution. Thus a memory reaction has a corresponding non-memory reaction in the non-memory time period. However, certain non-memory reactions such as (Eq. 2) may not be capable of firing during the memory time period.

To realize the firing capacity of different types of reactions, we introduced memory species that exist only in the memory time period. A chemical species is a normal species 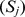 during the non-memory time period and may be a memory species 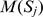 in the memory time period. For a memory reaction, at least one reactant and one product should be memory species; however, it is not necessary to define all species involving in a memory reaction as memory species. For example, the memory reaction for TF binding to the promoter site is represented by
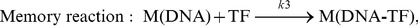
(3)where M(DNA) and M(DNA-TF) are memory species of DNA and DNA-TF, respectively. Thus the propensity functions of both memory reactions and non-memory reactions can be calculated simultaneously. Like the non-memory reaction, the memory reaction is also subject to stochastically distributed times between reaction instances. The time between reaction instances of both non-memory reaction and memory reaction can be determined in the same framework of the SSA.

Memory reactions normally are able to fire after a specific reaction occurs (e.g. the disassociation of RNAP from the promoter sites after the synthesis of the first transcript in a transcription cycle). This specific reaction is called the trigger reaction and its firing represents the start of a memory time period. Note that one trigger reaction may lead to two or more memory reaction time periods. When a trigger reaction fires, the finishing time points of the memory time periods are determined. The index of the memory reaction and finishing time point are stored in a queue structure that also saves the index and manifesting time point of delayed reactions.

A key issue in describing memory reaction is the transition between memory and non-memory species at the beginning and end of a memory time period. The firing of a trigger reaction transfers the normal species to the corresponding memory species. When a memory time period finishes, memory species should be transferred back to the normal species. Since memory species may involve in a number of memory reactions, the memory species may be free molecules 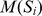, component of complexes including memory species (i.e. 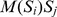), or compound of imaginary intermediate complex of delayed memory reactions. According to all the molecular complexes that contain the memory species, a number of transferring reactions should be defined for a memory reaction. When the memory time period finishes, these transferring reactions will be used to transfer the memory species back to the non-memory species.

### Memory stochastic simulation algorithm

The problem we are interested in is to simulate a well-stirred mixture of 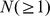 molecular species 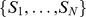 that chemically interact, inside some fixed volume Ω at a constant temperature, through 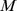 reactions 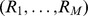, which include 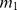 non-memory reactions, 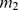 non-memory delayed reactions, 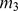 memory reactions, and 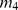 delayed memory reactions (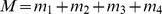). The system state is denoted as 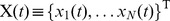, where 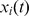 is the copy number of species 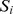 which is either a non-memory or memory species. We define a stoichiometric vector 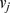 for either a non-memory or memory elementary reaction, consuming 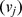 and manifest 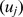 stoichiometric vectors for a non-memory or memory delayed reaction, as well as a number of stoichiometric vectors 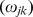 for transferring a memory species back to the corresponding normal species. For each reaction channel, a propensity function 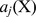 is defined and 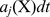 represents the probability of this reaction will fire inside Ω in the next infinitesimal time interval 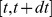. The memory stochastic simulation algorithm (memory-SSA) is given below.

Step 1. Set initial molecular numbers at 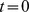, and an empty queue structure *L* for storing the information of delayed and memory reactions.
Step 2. Calculate propensity functions 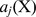, 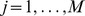, and 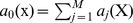.
Step 3. Generate a uniform random number 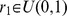 and determine the waiting time of the next reaction 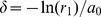.
Step 4. Compare 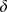 with the least time 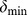 in the queue structure *L* to check whether there are delayed or memory reactions that are scheduled to finish within 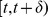.
Step 5. IF 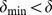

  IF (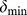 is associated with a non-memory or memory delayed reaction 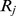)
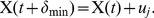
(4)
  ELSE (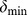 is associated with the finish of a memory time period)
    Find all the compounds with copy number 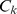 that include the memory species and use the corresponding stoichiometric vectors to update the system,
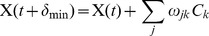
(5)
  ELSE:
    Determine the index 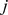 of the next reaction by a uniform random number 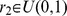


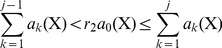
(6)and update the system state by
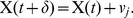
(7)
    If 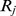 is a reaction with time delay 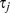, add the index 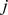 and updating time 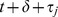 to the queue structure *L*.
    If 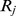 is a trigger reaction, add the memory index 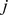 and finishing time 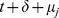 into the queue structure. Here 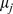 is the length of the memory time period.
Step 6. Go to Step 2.

To establish the theoretical foundation of the memory-SSA, we developed the memory chemical master equation and memory chemical Langevin equation. The memory chemical master equation include as special cases the delay chemical master equations [45] if memory reaction is not included in the system and the chemical master equation [46] if the chemical system comprises the elementary reactions only (see Supporting Information S1).

## Results

### Stochastic model for single-gene expression

**Figure 1:**
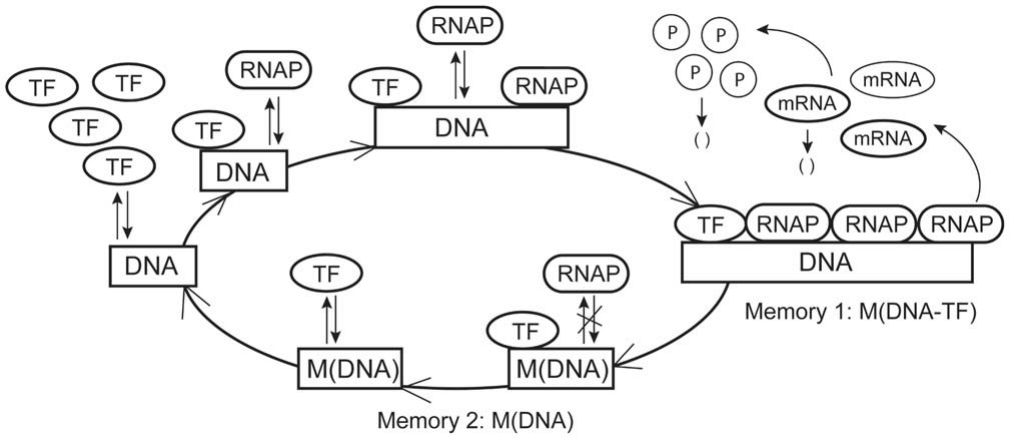
Regulatory network of a single gene. Regulatory mechanisms of gene expression include: binding of TF to a promoter site of the DNA; recruitment of RNAP to the promoter region to form the pre-initiation complex; binding of a number of RNAP molecules leading to multiple transcription re-initiations during a time period of gene activation, which is realized by the transcription memory window; gene inactivity period during which RNAP molecule is unable to bind to the promoter region, which is characterized as the second memory window.

To demonstrate the power of the proposed theory, a stochastic model with memory reactions was designed for single-gene expression for realizing the bursting expression dynamics (Fig. 1). The multitude of steps leading to an active transcription complex is represented by two major processes. First, a DNA with an unoccupied promoter site, to which RNAP is unable to bind, is activated by the binding of a TF to a specific response element in the promoter region. Then the TF acts as a platform to recruit the gene-specific regulators, represented by RNAP, to the local promoter region to form the pre-initiation complex, from which transcription can start. Once a successful preinitiation complex has been formed, reinitiation occurs with much higher probability. The activated transcription start site allows for the competitive binding of a number of RNAP molecules and multiple initiation events occur during one transcription cycle. The production of mRNA molecules per DNA template increased to a peak synthesis rate and then decayed rapidly because of an abrupt cessation of initiation [47]. Once a gene turns off, it takes quite a long time for the gene to be reactivated again, and no transcription occurs during this time period. Thus two memory time periods were designed to describe the continuous transcription and gene inactivity windows.

The transcription memory window was characterized by the memory complex M(DNA-TF) of the TF-DNA complex. The trigger reaction of this memory process of the first initiation of transcription
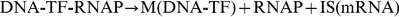
(8)where IS(mRNA) is the imaginary intermediate species to represent mRNA. The complex M(DNA-TF) recruits RNAP relatively faster than DNA-TF owing to the larger rate of transcription re-initiation; and the stability of the transcription pre-initiation complex leads to a burst of transcript production from the stable complex [6]. The end of the memory window for transcription is the start of the memory window of gene inactivity that was branded by the memory species M(DNA) of DNA (Eq. 3). In the inactivity window, the memory species M(DNA) can recruit TF to the operator site; however, it was assumed that the complex M(DNA)-TF cannot recruit RNAP and thus transcription was excluded from the gene inactivity window. This assumption is supported by experimental observations showing slow multistep sequential initiation mechanism for gene expression [47] and the relatively small numbers of multi-protein components of the transcriptional machinery [48]. The list of all chemical reactions was given in the Supporting Information S1 and detailed information of rate constants was provided in STable 1.

**Figure 2:**
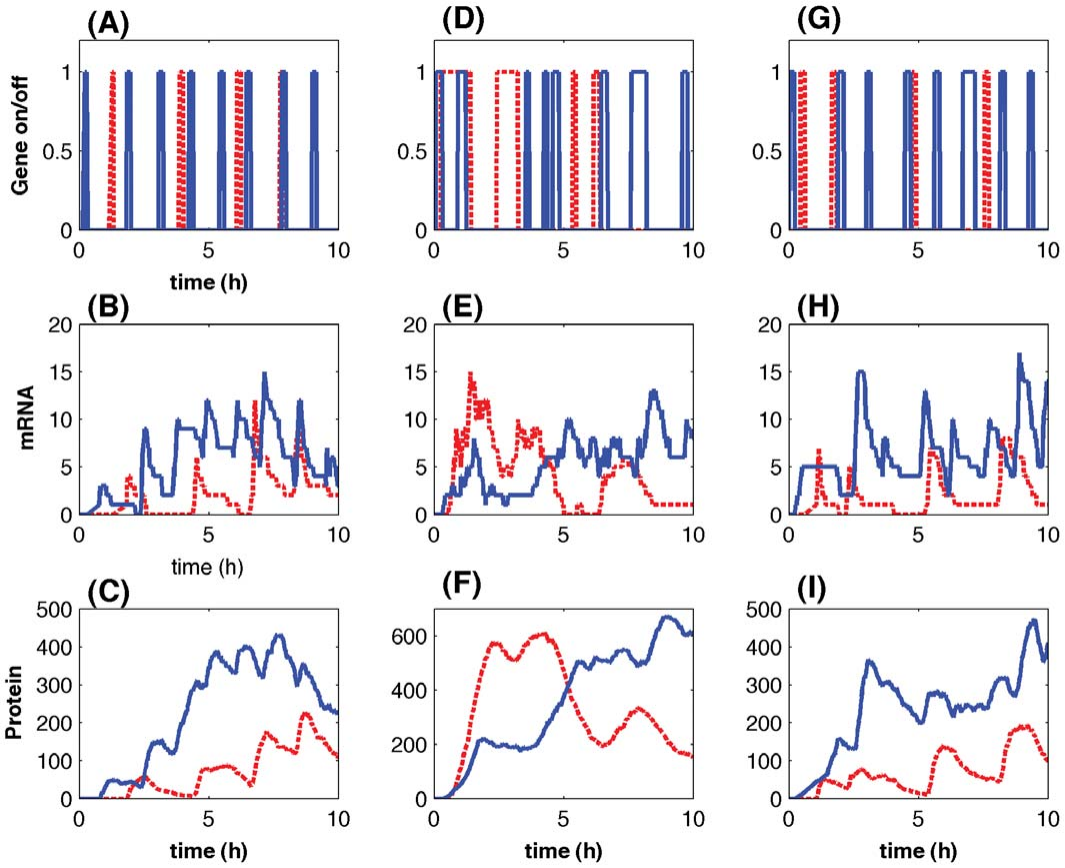
Stochastic simulations of single-gene expression using the same rate constants. (A) Gene On/Off states; (B) mRNA numbers; (C) protein numbers. Two simulations when the lengths of memory windows are constants (length of transcription window 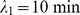 and length of gene inactivity window 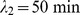). (D) Gene On/Off states; (E) mRNA numbers; (F) protein numbers. Two simulations when the lengths of memory windows follow the exponential distributions with mean 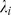. (G) Gene On/Off states; (H) mRNA numbers; (I) protein numbers. Two simulations when the lengths of memory windows follow the Gaussian distributions 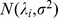 with 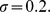

Fig. 2 gives simulations of the proposed model using the same rate constants but the lengths of memory windows follow different distributions. Here we are particularly interested in the exponential distribution that has been used to generate the waiting times between two consecutive gene expression cycles. When the lengths of memory windows are constant in Fig. 2A, 2B and 2C, the disparity between the number of transcripts synthesized in different bursts is not large. However, the variation of mRNA copy numbers in different expression cycles is large in Fig. 2E if the lengths of memory windows follow the exponential distributions. The large variation of the transcript numbers leads to large variation in protein copy numbers in Fig. 2F. We also used the Gaussian random variables to generate samples for the length of memory windows. Simulations in Figure 2G, 2H and 2I suggested that the variation of mRNA copy numbers in different expression cycles is larger than that using constant lengths of memory windows but smaller than that when the length of memory windows follows the exponential distribution.

**Figure 3:**
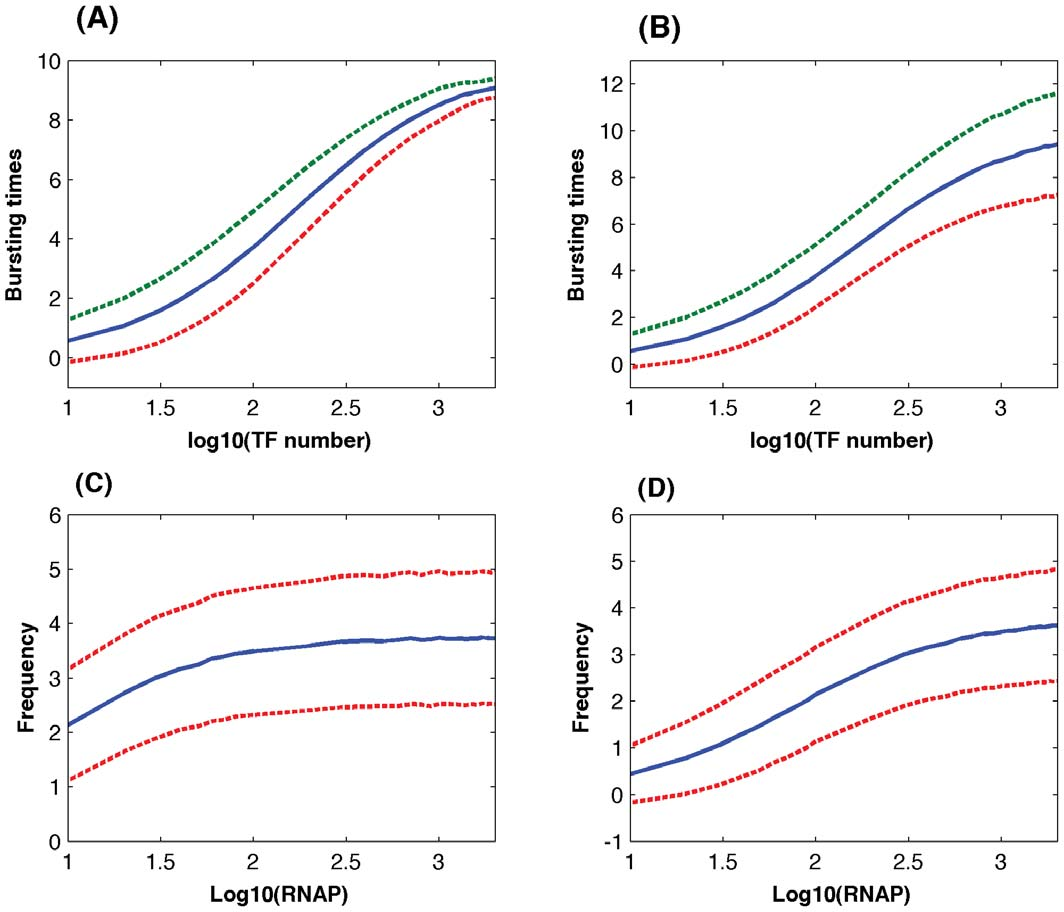
Averaged bursting numbers under various conditions. The averaged bursting number per simulation based on different numbers of TF but a fixed number of RNAP with either constant lengths of memory windows in (A) or lengths following the exponential distributions in (B). Rate constant are the same as those in Figure 2. The averaged bursting number per simulation based on different numbers of RNAP but a fixed TF number with the binding rate of RNAP to DNA as 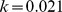 in (C) or 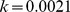 in (D). The corresponding rate constant in Figure 2 is 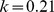 (solid line: mean; dash-line: 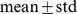).

To find the factors determining the frequency of transcription cycles, simulation results were obtained by using different TF numbers but a fixed RNAP number (Figs. 3A and 3B). When the lengths of memory time periods follow the exponential distributions, the averaged bursting number in Fig. 3B is slightly larger than or equal to that in Fig. 3A where the lengths of memory time periods are constants. When the TF numbers are not large 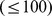, both the averaged bursting number and standard deviation in Fig. 3A and 3B are very close to each other. However, if the TF number is large 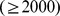, the standard deviation of the simulations using the exponential distributions is much larger than that obtained from simulations with constant length of memory time periods. We further simulated the stochastic model using a fixed number of TFs, but different RNAP numbers together with different binding rate constants of RNAP molecules to the DNA-TF complex (Fig. 3C and 3D). Simulation results in Fig. 3 suggested that the probability to form the initiation complex is strongly correlated with the frequency of transcription. In the proposed model, TF and RNAP are two symbolic species to represent the transcriptional machinery and promoter factors. Thus these results are in good agreement with the experimental observations showing that the factors initiating gene transcription are the primary regulatory mechanisms to determine the frequency of transcriptional cycles [49].

**Figure 4:**
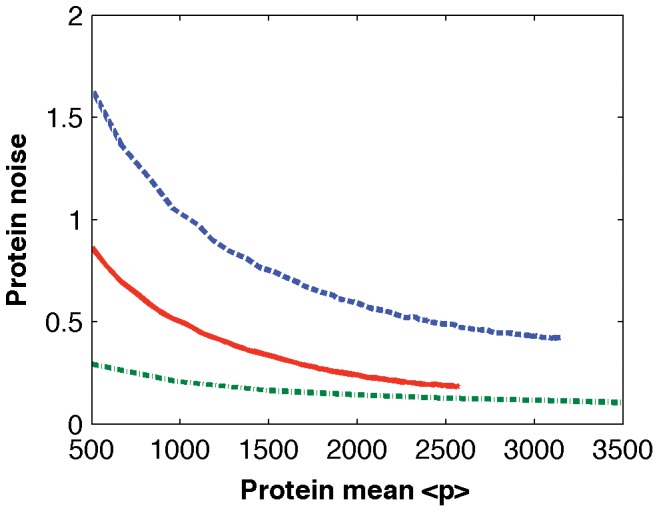
Simulated noise in protein abundance. Noise in protein abundance 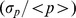 derived from stochastic simulations with different TF numbers (solid-line: lengths of memory windows are constant; dash-line: lengths of windows follow the exponential distributions; dash-dot line: theoretical prediction from a simpler stochastic model in [19]).

One of the major results derived from a stochastic model of the single-gene network is that the noise in protein abundance is anti-proportional to the averaged protein copy number [19]. Thus an important question is whether this theoretical finding derived from a simpler stochastic model still holds when more detailed dynamics of gene expression is considered in this work. To answer this question, we calculated noise in protein abundance based on stochastic simulations with different TF numbers. The simulated noise in protein abundance derived from 10,000 simulations for each TF number was plotted against the averaged protein numbers. When the lengths of memory windows are constant, Fig. 4 shows that the simulated noise is larger than but proportional to the theoretical prediction in [19]. Furthermore, the simulated noise is even larger if the lengths of memory windows follow the exponential distributions. Thus our simulation results are in good agreement with the theoretical finding. It is reasonable to expect that the noise in protein abundance is larger if more random resources are considered.

### Stochastic model of the p53-MDM2 core module

**Figure 5:**
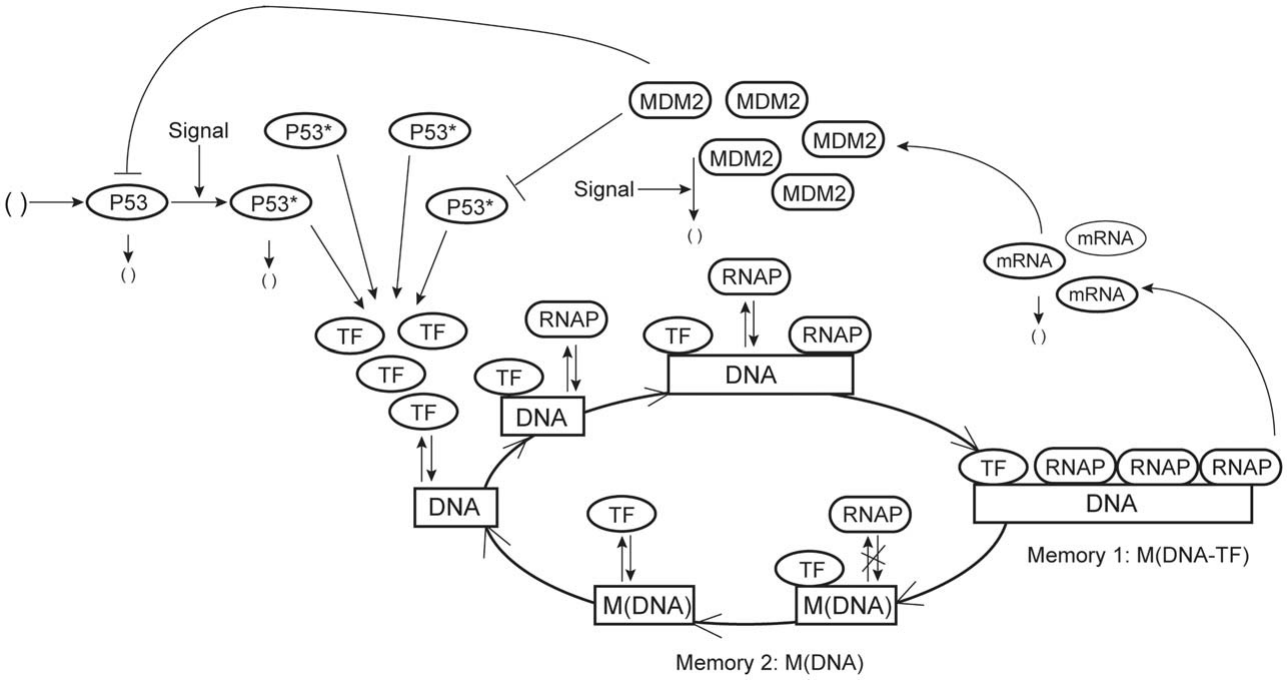
The p53-MDM2 core module. P53 protein is activated by the upstream signal (represented by ATM kinase) and form tetramers as the TFs. p53 positively regulates gene *MDM2* by activating its transcription, whereas MDM2 negatively regulates p53 by promoting its ubiquitination and degradation. Regulatory mechanisms for the expression of gene *MDM2* follow the same assumptions in Figure 1, which are characterized by the two memory windows for the continuous transcription and inactivity time periods of gene *MDM2*.

The success in realizing the bursting gene expression stimulated us to go one step further to examine the mechanisms regulating the p53 core module (Fig. 5). Under normal unstressed conditions the negative regulation of MDM2 keeps p53 activity at low levels; but under various stress conditions, upstream mediators such as ATM and Chk2 kinases are activated and induce post-translational modification on p53 and MDM2 [50]. These modifications lead to stabilization of p53 and an increase in p53 activity. Experimental studies in populations of cultured cells showed that p53 and MDM2 undergo damped oscillatory behavior following DNA damage caused by gamma irradiation [51]. However, the protein dynamics observed in single cells was similar to digital clock behavior [9], [52]. Although mathematical models have been designed to simulate the network dynamics either at population level [50], [51], [53], [54] or at single-cell level [50], [52], [55], it is still a challenge to realize experimental observations in single cells and population of cells simultaneously [56].

**Figure 6:**
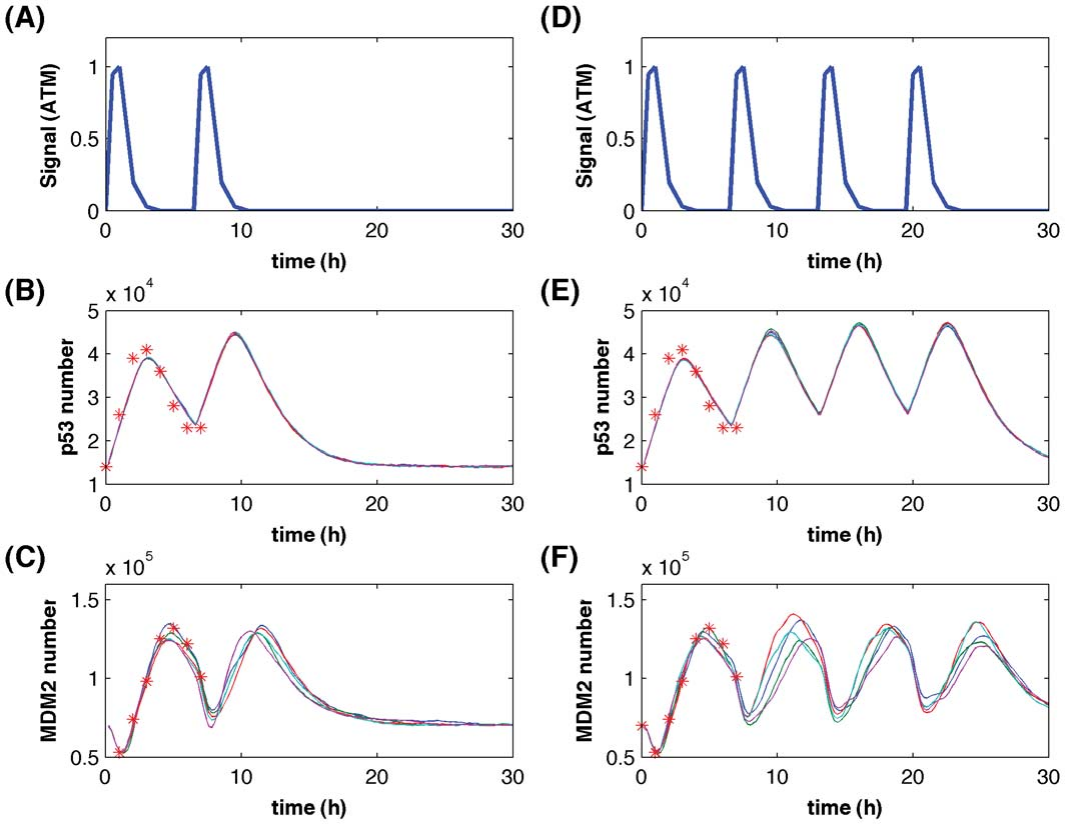
Stochastic simulations of the p53-MDM2 core module. The upstream signal represented by the ATM kinase activities (measured from Fig. 1 in [50]) has two pulses in (A) or four pulses in (D). Five simulations of the p53 copy numbers based on two pulses (B) and four pulses (E) of the upstream signal; and the corresponding MDM2 copy numbers in five simulations induced by two pulses (C) and four pulses (F) of p53 activities.

To tackle this challenge, a stochastic model with memory reactions (see Supporting Information S1) was designed to describe the dynamics of the p53 core circuit using rate constants estimated from experimental data that were given in STable 2. The transcription process of MDM2 follows the same assumptions in Fig. 1. We used two memory reactions to represent the gene activation and inactivation windows. Following experimental observations, it was assumed that the expression of gene MDM2 is activated continuously over a period of ∼1 h and then an inactivated window of ∼5.5 h follows [9]. Using the activity of ATM kinase as the upstream signal [50], Fig. 6 gives simulated protein numbers of p53 and MDM2 that were activated by the upstream signal with different pulse numbers. Simulations precisely realized experimentally measured p53 and MDM2 molecular numbers [57]. The sustained upstream signal maintained continuous oscillations of p53 activity that led to the corresponding expression cycles of gene *MDM2*. Simulations suggested that the feedback regulations between p53 and MDM2 are not sufficient to continue the expression oscillations. The p53 activities gradually return to the basal levels after one expression cycle if the upstream signal ceases. When the p53 activity is below a threshold value, the TF activity is not adequate to stimulate another expression cycle of gene *MDM2*. Although the decrease of MDM2 activity contributes to the accumulation of p53 proteins, this negative regulation is not critical for the increase of the p53 transcriptional activity.

**Figure 7:**
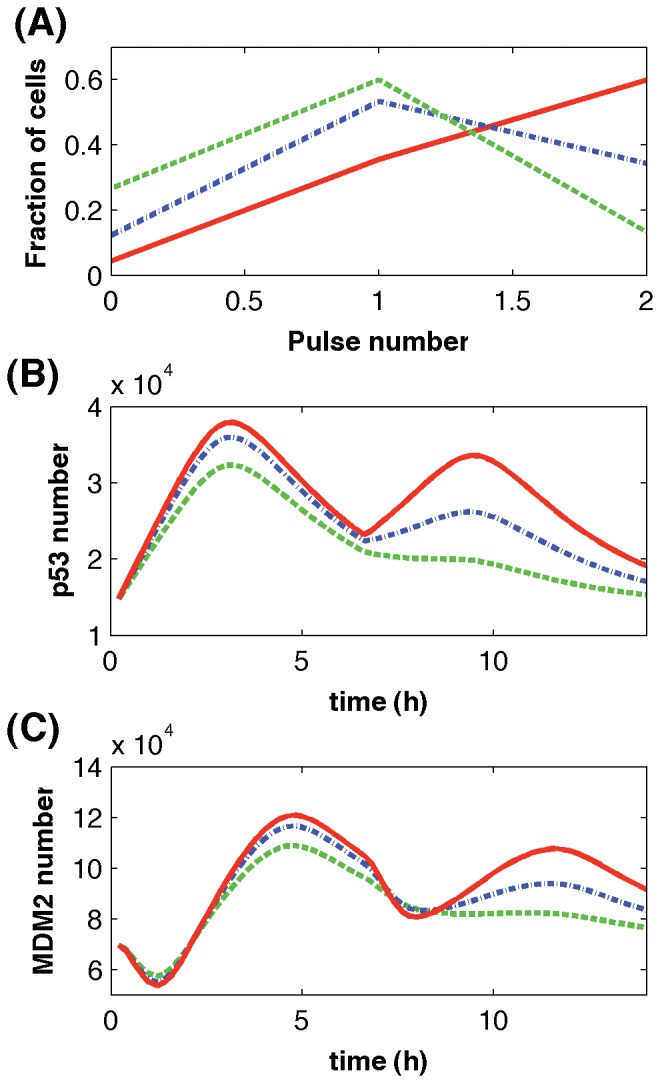
Damped oscillation of the p53 module in a population of cells. (A) Fractions of cells showing different pulse numbers of ATM activity when cells were irradiated by different gamma doses. The averaged copy numbers of p53 (B) and MDM2 (C) based on 1000 simulations. (Solid-line: gamma dose 10 Gy, dash-dot-line: 2.5 Gy, and dash-line: 0.3 Gy).

We have demonstrated that the proposed gene activation window play a key role in inducing gene expression bursts with fairly constant width and height at the single cell level. The next question is whether the proposed stochastic model can realize the damped oscillations observed at population level. To answer this question, stochastic simulations were obtained by using different pulse numbers of the upstream signal in different simulations. According to simulations in Figs. 6B and 6E, it was assumed that the pulse number of the upstream signal was equal to the p53 pulse number. Thus the fraction of cells with different pulse numbers of the upstream signal in Fig. 7A is the same as that of the p53 pulse numbers which was estimated from Fig. 3 in [9]. Simulations in Figs. 7B and 7C successfully realized the damped oscillations of p53 and MDM2 protein levels that were compatible to experimental observations [51]. The height of oscillations at population level is proportional to the dose of gamma radiation. Simulations suggested that a higher radiation dose induced a larger fraction of cells showing more pulses of p53 activity, which led to the higher expression levels of gene *MDM2* at population level in Figure 7C.

## Discussion

This work proposed the concept of memory reaction to describe conditional chemical reactions that occur in the path of memory events. The proposed memory-SSA represents an innovative strategy to use a reduced model to describe nonlinear dynamics. To demonstrate the power of the proposed theory, we developed a stochastic model of single-gene expression. Numerical simulations suggested that memory reactions for realizing gene activation/inactivation windows play a major role in generating bursting dynamics of gene expression. The function of memory reactions has been further supported by realizing the oscillatory activities of the p53 core module in single cells. Simulations suggested that memory process is a key mechanism to generate sustained oscillations of protein levels in single cells and damped oscillations in population of cells. These successful applications suggested that the proposed theory is an effective tool to realize conditional chemical reactions in a wide range of complex biological system.

Time delay is a modeling technique to realize slow reactions or simplify multiple small step reactions [24], [25]. It is emphasized that the difference between the delayed reaction and the proposed memory reaction is substantial. First, the firing of delayed reactions depends on the competition with other reactions in the system. However, the occurrence of memory reactions is conditional to the path of memory events, though simultaneously the firing of memory reactions also depends on the competition with other reactions if it is within the memory time period. In addition, the key feature of delayed reaction is the time difference between the firing of a chemical reaction and manifest of its products. However, the products of a memory reaction are generated immediately after its firing. In this work we also proposed the delayed memory reaction if the reaction is conditional to the path of memory events as well as there is delay between the firing of the chemical reaction and manifest of its products. Furthermore, molecules involving in delayed reactions are static during the delayed time period because they are reserved for the product manifest in a future time point; however, molecules involving in memory reactions are dynamic since they involve in other reactions in the memory window. Thus the memory and time delay are two distinct features of chemical reactions, though these two types of reactions are connected to a fixed length of time period.

Regarding the necessary of memory reactions, one may argue that the memory phenomena may be simply realized by using additional species and additional chemical reactions within the classic SSA framework. If this modeling scheme were implemented without using memory reactions, the competitive nature of the elementary stochastic chemical reactions would cause that the time period of a particular biological/cellular event does not follow the distribution observed in experiments. For example, the rapid re-initiation rate of transcription should be matched by a large termination rate of gene expression, namely the rate of TF disassociating from the DNA promoter site. In this case the exit strategy of gene expression is realized by the competitive reaction of TF disassociation. However, our simulation results suggested that it is difficult to use this strategy to realize the relatively constant time periods of gene expression that were observed in experiments. In this work we proposed the memory reaction to realize such refractory states that exist only in a particular time period. The key feature of the memory reaction is the exit strategy for determining the length of memory time period and for defining exit reactions for transferring memory species to the normal species. There are two time periods that are associated with memory reactions, namely the waiting time for the firing of a memory reaction and the memory time period during which memory reactions are capable of firing. Although the waiting time of memory reaction still follows an exponential distribution, the length of a memory time period can be defined as a constant or a random variable following a particular distribution, such as the Gaussian or exponential distribution. By properly defining the length of memory time period, we have successfully realized the stochastic dynamics of biological networks that also have certain deterministic feature. Therefore, the proposed memory reaction represents a quantum step towards the development of sophisticated modeling methodologies to explore the regulatory mechanisms of complex biological systems.

Although different modeling approaches have been proposed to realize noisy process in gene expression [58], [59], [60], recent experimental observations suggested that the expression dynamics has certain deterministic properties including the relatively constant heights and durations of expression bursts. These stochastic events may be regulated by complex networks that are still not fully understood; or the underlying mechanisms may be too complex to be represented by reduced mathematical models. These mechanisms may include the chromatin modification and chromatin looping formation, the spatio-temporal dynamics of protein movement, as well as the intrinsically cyclic association of transcriptional factors and their co-factors. It may not be practical to use competitive chemical reactions in the SSA or delay-SSA framework to represent these stochastic events with deterministic properties. To this end, the proposed memory reaction provides a powerful tool to describe the complex regulatory mechanisms by using reduced mathematical models. In addition, it is expected that memory reaction will be used as a mechanism to realize the robustness property of biological systems [61], [62].

The gene activation and inactivation windows realized by memory reaction provided novel insight into the origin of the repeated pulses in the p53-MDM2 core module. In particular, the stable time periods of gene activation play a major role in generating bursting dynamics with constant width and height of protein activity oscillations. A striking simulation result is that the oscillatory upstream signal is the key stimulus to maintain oscillatory dynamics of the p53 core module. In contract, the feedback regulations between p53 and MDM2 are not sufficient to maintain the oscillations of the p53 activity. This result is well compatible with the recent experimental observations showing that p53 induction is mediated by the damage-activated regulators [50], [63]. Since a number of important regulatory mechanisms were excluded from the proposed stochastic model, including protein spatial distributions, regulation of other proteins such as MDMX, and feedback regulations between the upstream signals, more sophisticated models are needed to provide accurate simulations and testable predictions.

## Supporting Information

Supporting Information S1 **A detailed description of the memory stochastic simulation algorithm (memory-SSA); the theory of the memory chemical master equation and memory stochastic differential equations; assumptions, chemical reactions and rate constants of a stochastic model with memory reactions for describing the expression of a single gene and for the stochastic model of the p53-MDM2 regulatory network.**
(PDF)
