# Supplementary material for: Chemical Memory Reactions Induced Bursting Dynamics in Gene Expression
Source: PLoS One. 2013 Jan 21;8(1):e52029. doi: 10.1371/journal.pone.0052029 (PMC3549921; doi:10.1371/journal.pone.0052029)
Supplement: Supporting Information S1 — A detailed description of the memory stochastic simulation algorithm (memory-SSA); the theory of the memory chemical master equation and memory stochastic differential equations; assumptions, chemical reactions and rate constants of a stochastic model with memory reactions for describing the expression of a single gene and for the stochastic model of the p53-MDM2 regulatory network. (PDF) [file pone.0052029.s001.pdf]

Supporting Information S1

Chemical memory reactions induced bursting dynamics in gene expression

Tianhai Tian

This supplementary information first gives a detailed description of the memory stochastic simulation algorithm (memory-SSA) for simulating biological systems with memory in section 1. Section 2 derives the theory of the memory chemical master equation and memory stochastic differential equations. Section 3 lists the assumptions, chemical reactions and rate constants of a stochastic model with memory reactions for describing the expression of a single gene. Section 4 provides detailed information regarding the assumptions, chemical reactions and rate constants for the stochastic model of the p53-MDM2 regulatory network.

1. Memory stochastic simulation algorithm (memory-SSA)

It is assumed that a well-stirred chemical reaction system contains N molecular species $\{S_1, S_2, \ldots, S_N\}$ with number $x_i(t)$ of the species S_i at time t. The system state is denoted as $X(t) \equiv \{x_1(t), \ldots x_N(t)\}^{T}$. It is also assumed that the system contains M reaction channels $(R_1, \ldots, R_M)$. Reactions are classified into two categories, namely the non-memory reactions (or briefly reactions) and memory reactions, and each category includes elementary reactions and delayed reactions.

1.1. Elementary reaction

Elementary reactions produce products immediately after reactants are consumed. For example, an elementary reaction consuming reactants S_i and S_j and producing products S_k and S_l is given by

$$S_i + S_j \xrightarrow{r} S_k + S_l, \quad (1.1)$$

where r is the rate constant. A stoichiometric vector v_j is defined for this reaction to represent the change of molecular numbers due to the firing of this reaction. It is assumed that the waiting time to the next reaction follows an exponential distribution. The stochastic simulation algorithm (SSA) was designed to simulate chemical systems with elementary reactions only [1].

1.2. Delayed reaction

Delayed reactions consume reactants instantly but it takes a certain time period to generate the products due to the time delay. For example, a delayed reaction with time delay τ, which consumes reactants S_i and S_j as well as produces product S_k and S_l, is represented by

$$S_i + S_j \xrightarrow{k} U[+S_i + S_j], \quad (1.2)$$

$$U \to S_k + S_l, \quad (1.3)$$

where U is an imaginary intermediate species existing during the delayed time period. Reaction (1.2) occurs instantaneously; and its waiting time δ follows an exponential distribution. The product manifest (1.3) takes place at $t+\delta+\tau$, where t is the current time point. This delayed reaction may be either a non-consuming delayed reaction, if S_i and S_j appear on the right-hand side of reaction (1.2), or a consuming delayed reaction if S_i or S_j or both is absent on the right-hand side of reaction (1.2). Two stoichiometric vectors v_j and u_j are respectively designed for the consuming reaction (1.2) and product manifest (1.3). Both the consuming and non-consuming delayed reactions can be represented in a single framework by properly designing the stoichiometric vectors v_j. Therefore we do not distinguish between these two types of delayed reactions. The delay stochastic simulation algorithm (delay-SSA) was designed to simulate chemical reaction systems with time delay [2, 3].

1.3. Memory reaction

A memory reaction fires during the path of a molecular memory event. It is realized by a chemical reaction that occurs during a particular time-period and/or under specific system conditions. A memory reaction may be either an elementary reaction or a delayed reaction. The time period during which memory reactions may fire is termed as **the memory time period**. The length of a memory time period may be either a constant or a random variable with an associated probability distribution. A memory reaction system may have several memory time periods. If the overlap between the time periods is small, it can be assumed that the probability distributions of the lengths of these time periods. Otherwise, a joint distribution should be defined to determine the length of each memory time period.

An example of the memory events is the refractory time period during which an organ or cell is incapable of repeating a particular action. In gene expression, one of the refractory states is the chromatin epigenetic process, such as silencing by DNA methylation and structural changes in chromatin [39,40]. Since silencing molecules are recruited by an autocatalytic mechanism, this can lead to a long periods of reactivation. During the time period of transcriptional activation, both the transcriptional factor (TF) and RNA polymerase (RNAP) can bind to the corresponding promoter site, which has been modeled by the following elementary reactions

Elementary reaction: DNA + TF $\xrightarrow{k1}$ DNA-TF (1.4)

Elementary reaction: DNA-TF + RNAP $\xrightarrow{k2}$ DNA-TF-RNAP (1.5)

However, during the refractory period, the transcriptional activators could gain access to silenced chromatin but that RNAP and TATA-binding protein (TBP) are excluded [43,44]. Therefore reaction (Eq. 1) may fire but reaction (Eq. 2) is unable to fire during the silencing time period. A new reaction is needed to realize the event in the refractory period. Such reaction is defined as memory reaction in this work. Thus a memory reaction has a corresponding non-memory reaction in the non-memory time period. However, certain non-memory reactions such as (1.5) may not be capable of firing during the memory time period.

A chemical species is a normal species (S_j) during the non-memory time period and may be a **memory species** $M(S_j)$ in the memory time period. For a memory reaction, at least one reactant and one product should be memory species; however, it is not necessary to define all species involving in a memory reaction as memory species. Since all memory species should return to the normal species when the memory time period finishes, more memory species may lead to a more complex process to transfer variables between normal species and memory species. For example, the memory reaction for TF binding to the promoter site is represented by

$$\text{Memory reaction: M(DNA) + TF} \xrightarrow{k3} \text{M(DNA-TF)}, \quad (1.6)$$

where M(DNA) and M(DNA-TF) are memory species of DNA and DNA-TF, respectively. The propensity function of memory reaction (1.6) is

$$a_j(\mathrm{X}) = k_3[M(DNA)]*[TF]. \quad (1.7)$$

Therefore the propensity functions of both memory reactions and non-memory reactions are calculated simultaneously. However, the copy numbers of memory species are zero during the non-memory time period, and in this way memory reactions cannot fire during the non-memory time period.

1.4 Delayed memory reaction

A chemical reaction is a delayed memory reaction if it fires during the path of a memory event and also takes certain time for product manifest. Using the delayed reaction (1.2, 1.3) as an example, if species S_i and S_k have the corresponding memory species $M(S_i)$ and $M(S_k)$, respectively, the corresponding memory delayed reaction is represented by

$$M(S_i) + S_j \xrightarrow{k} M(U)[+S_i + S_j], \quad (1.8)$$

$$M(U) \rightarrow M(S_k) + S_l. \quad (1.9)$$

where $M(U)$ is the imaginary memory species during the period of time delay.

If the time point of product manifest is within the memory time period, the imaginary memory species $M(U)$ will be updated by reaction (1.9) directly. However, if the memory time period finishes before the product manifest, the imaginary memory species will be updated by

$$M(U) \to U \tag{1.10}$$

when the memory time period finish, which is implemented by changing the index of this delayed reaction. The update will be carried out by reaction (1.3) at the time point of product manifest.

1.5 Trigger reactions and Transition between non-memory and memory species

Memory reactions normally are able to fire after a specific reaction occurs (e.g. the disassociation of RNAP from the promoter sites after the synthesis of the first transcript in a transcription cycle). This specific reaction is called the trigger reaction and its firing represents the start of a memory time period. Note that one trigger reaction may lead to two or more memory reaction time periods. When a trigger reaction fires, the finishing time points of the memory time periods are determined. The index of the memory reaction and finishing time point are stored in a queue structure that also saves the index and manifesting time point of delayed reactions.

A key issue in describing memory reaction is the transition between memory and non-memory species at the beginning and end of a memory time period. The firing of a trigger reaction transfers the normal species to the corresponding memory species. When a memory time period finishes, memory species should be transferred back to the normal species. Since memory species may involve in a number of memory reactions, the memory species may be free molecules $M(S_i)$, component of complexes including memory species (i.e. $M(S_i)S_j$), or compound of imaginary intermediate complex of delayed memory reactions. According to all the molecular complexes that contain the memory species, a number of transferring reactions should be defined for a memory reaction. When the memory time period finishes, these transferring reactions will be used to transfer the memory species back to the non-memory species. For example, to transfer $M(S_i)$ back to the species S_i, we may consider the following three-updating reactions

Free memory species: $M(S_i) \to S_i$ (1.11)

Compound with memory species: $M(S_i)S_j \to S_i + S_j$ (1.12)

Imaginary memory species: $M(U) \to U$ (1.13)

As discussed in Section 1.4, we only need to change the index of the delayed reaction in the queue structure if reaction (1.13) is used.

1.6 Memory stochastic simulation algorithm

The problem we are interested in is to simulate a well-stirred mixture of $N(\geq 1)$ molecular species $\{S_1,\ldots,S_N\}$ that chemically interact, inside some fixed volume Ω at a constant temperature, through M reactions $(R_1,\ldots,R_M)$, which include m_1 non-memory reactions, m_2 non-memory delayed reactions, m_3 memory reactions, and m_4 delayed memory reactions ($M = m_1 + m_2 + m_3 + m_4$). The system state is denoted as $\mathrm{X}(t) \equiv \{x_1(t),\ldots x_N(t)\}^{\mathrm{T}}$, where $x_i(t)$ is the copy number of species S_i which is either a non-memory or memory species. We define a stoichiometric vector v_j for either a non-memory or memory elementary reaction, consuming (v_j) and manifest (u_j) stoichiometric vectors for a non-memory or memory delayed reaction, as well as a number of stoichiometric vectors (ω_{jk}) for transferring a memory species back to the corresponding normal species. For each reaction channel, a propensity function $a_j(\mathrm{X})$ is defined and $a_j(\mathrm{X})dt$ represents the probability of this reaction will fire inside Ω in the next infinitesimal time interval $[t, t+dt]$. The memory-SSA is given below.

Step 1. Set initial molecular numbers at t = 0, and an empty queue structure L for storing the information of delayed and memory reactions.

Step 2. Calculate propensity functions $a_j(\mathrm{X})$, $j = 1,\ldots,M$, and $a_0(\mathrm{x}) = \sum_{j=1}^{M} a_j(\mathrm{X})$.

Step 3. Generate a uniform random number $r_1 \in U(0,1)$ and determine the waiting time of the next reaction $\delta = -\ln(r_1)/a_0$.

Step 4. Compare δ with the least time $\delta_{\min}$ in the queue structure L to check whether there is any delayed or memory reactions that are scheduled to finish within $[t, t+\delta)$.

Step 5. IF $\delta_{\min} < \delta$

IF ($\delta_{\min}$ is associated with a non-memory or memory delayed reaction R_j)

$$\mathrm{X}(t+\delta_{\min}) = \mathrm{X}(t) + u_j. \tag{1.14}$$

ELSE ($\delta_{\min}$ is associated with the finish of a memory time period)

Find all the compounds with copy number C_k that include the memory species and use the corresponding stoichiometric vectors to update the system,

$$\mathrm{X}(t+\delta_{\min}) = \mathrm{X}(t) + \sum_j \omega_{jk} C_k \tag{1.15}$$

ELSE:

Determine the index j of the next reaction by a uniform random number $r_2 \in U(0,1)$

$$\sum_{k=1}^{j-1} a_k(\mathrm{X}) < r_2 a_0(\mathrm{X}) \le \sum_{k=1}^{j} a_k(\mathrm{X}) \quad (1.16)$$

and update the system state by

$$\mathrm{X}(t+\delta) = \mathrm{X}(t) + v_j . \quad (1.17)$$

If R_j is a reaction with time delay τ_j, add the index j and updating time $t+\delta+\tau_j$ to the queue structure L.

If R_j is a trigger reaction, add the memory index j and finishing time $t+\delta+\mu_j$ into the queue structure. Here μ_j is the length of the memory time period.

Step 6. Go to Step 2.

2. Memory chemical master equation

To compare the delay-SSA and the proposed memory-SSA, this section first derives the memory chemical master equation for biological systems with memory reactions but without any delayed reaction. Next we will design the chemical master equation for biological systems with both delayed and memory reactions.

2.1 Memory chemical master equation for systems without delayed reactions

Following the notations defined in Section 1.6, we consider a biological system with m non-memory reactions ($R_1,\ldots,R_m$) and $M-m$ memory reactions ($R_{m+1},\ldots,R_M$). For each memory reaction R_j ($j \in \{m+1,\ldots,M\}$), there is a corresponding trigger reaction R_j^* whose propensity function is denoted as $a_j^*(\mathrm{X})$. The memory time period during which R_j may fire has length μ_j. The trigger reaction is a non-memory reaction and $a_j^*(\mathrm{X})$ is one of the propensity functions a_i ($i \in \{1,\ldots,m\}$). Note that a trigger reaction starts a memory time period that may include a number of memory reactions.

We are interested in the probability function of the system state $\mathrm{X}(t)$ at time t, and will derive the time evolution equation of the probability function

$$P(\mathrm{X},t) \equiv \mathrm{Prob}\{\mathrm{X}(t) = \mathrm{X} \mid \mathrm{X}(t_0) = \mathrm{X}_0\} . \quad (2.1)$$

For a biochemical system with memory reactions, the master equation should be based on the current system state at time t for the non-memory reactions, and the system states during the interval $[t-\mu_j, t]$ for a memory reaction R_j whose memory time period has length μ_j. We take a time increment dt that is so small that the probability for two or more reactions to occur in dt is

negligible compared to the probability for at most one reaction. It is assumed that the time of a reaction is dt. Then the probability of the system being in the state x at $t+dt$ is given by

$$\begin{aligned}
P(\mathrm{X},t+dt) &= P(\mathrm{X},t)-\sum_{j=1}^{m} a_j(\mathrm{X})P(\mathrm{X},t)dt+\sum_{j=1}^{m} a_j(\mathrm{X}-v_j)P(\mathrm{X}-v_j,t)dt \\
&-\sum_{j=m+1}^{M}\sum_{k=0}^{Nj-1}\sum_{\mathrm{X}_k\in I(\mathrm{X})} a_j(\mathrm{X})dtP(\mathrm{X},t;\mathrm{X}_k,t_k)a_j^*(\mathrm{X}_k)(t_{k+1}-t_k) \\
&+\sum_{j=m+1}^{M}\sum_{k=0}^{Nj-1}\sum_{\mathrm{X}_k\in I(\mathrm{X})} a_j(\mathrm{X}-v_j)dtP(\mathrm{X}-v_j,t;\mathrm{X}_k,t_k)a_j^*(\mathrm{X}_k)(t_{k+1}-t_k) \\
&-\sum_{j=m+1}^{M}\sum_{\mathrm{X}_i\in I(\mathrm{X})} a_j^*(\mathrm{X}_i)P(\mathrm{X},t;\mathrm{X}_i,t-\mu_j)dt+\sum_{j=m+1}^{M}\sum_{\mathrm{X}_i\in I(\mathrm{X})} a_j^*(\mathrm{X}_i)P(\mathrm{X}-\omega_j,t;\mathrm{X}_i,t-\mu_j)dt
\end{aligned} \tag{2.2}$$

where $P(\mathrm{X},t;\mathrm{X}_k,t_k)$ is the probability that the system is in the state X at time t and also in the state X_k at t_k, and $I(\mathrm{X})$ is the set of all possible system states. In addition, $t-\mu_j=t_0<t_1<\ldots<t_{Nj}=t$ is a partition of the interval $[t-\mu_j,t]$. The second and third items on the right hand side of equation (2.2) describe the change of system states due to the firing of non-memory elementary reactions. The fourth and fifth terms describe the change of system states because of the firing of memory reactions. The memory reaction R_j is capable of firing after the corresponding trigger reaction R_j^* fires during any time interval $[t_k,t_{k+1}]\subset[t-\mu_j,t]$. Thus the probability for the firing of a memory reaction is given by

$$P=a_j(\mathrm{X})dtP(\mathrm{X},t;\mathrm{X}_k,t_k)a_j^*(\mathrm{X}_k)(t_{k+1}-t_k) \tag{2.3}$$

that means at time t_k the trigger reaction R_j^* fires during $[t_k,t_{k+1}]$ and at time t reaction R_j fires. The last two terms in (2.2) describe the transfer of memory species back to normal species. The stoichiomechic vector of the species transfer is ω_j. For simplicity, it was assumed that the copy number of free memory species is always positive, and thus only one stoichiometric vector ω_j is used in (2.2).

If the length μ_j of the memory time period is large and there is a relatively large number of reactions firing in the time interval $[t-\tau_j,t)$, it is assumed that the coupling of the system states at t and t_k is weak, namely

$$P(\mathrm{X},t;\mathrm{X}_k,t_k)\approx P(\mathrm{X},t)P(\mathrm{X}_k,t_k). \tag{2.4}$$

It means the firing of the memory reaction R_j is independent of the firing of the triggering reaction R_j^*. When $dt\to 0$, this lead to the memory chemical master equation

$$\frac{\partial}{\partial t}P(\mathrm{X},t) = -\sum_{j=1}^{m} a_j(\mathrm{X})P(\mathrm{X},t) + \sum_{j=1}^{m} a_j(\mathrm{X}-\upsilon_j)P(\mathrm{X}-\upsilon_j,t)$$
$$- \sum_{j=m+1}^{M} a_j(\mathrm{X})P(\mathrm{X},t)\int_{t-\mu_j}^{t} \overline{a_j^*(\mathrm{X}(s))}ds + \sum_{j=m+1}^{M} a_j(\mathrm{X}-\upsilon_j)P(\mathrm{X}-\upsilon_j,t)\int_{t-\mu_j}^{t} \overline{a_j^*(\mathrm{X}(s))}ds \qquad (2.5)$$
$$- \sum_{j=m+1}^{M} \overline{a_j^*(\mathrm{X}(t-\mu_j))}P(\mathrm{X},t) + \sum_{j=m+1}^{M} \overline{a_j^*(\mathrm{X}(t-\mu_j))}P(\mathrm{X}-\omega_j,t)$$

where the mean of the propensity function of the triggering reaction is

$$\overline{a_j^*(\mathrm{X}_k)} = \sum_{\mathrm{X}_k \in I(\mathrm{X})} a_j^*(\mathrm{X}_k)P(\mathrm{X}_k,t_k) \qquad (2.6)$$

and the integration in (2.5) is derived from

$$\begin{aligned}\int_{t-\mu_j}^{t} \overline{a_j^*(\mathrm{X}(s))}ds &= \lim_{Nj\to\infty} \sum_{k=0}^{Nj-1} \overline{a_j^*(\mathrm{X}_k)}(t_{k+1}-t_k) \\ &= \lim_{Nj\to\infty} \sum_{k=0}^{Nj-1} \sum_{\mathrm{X}_k\in I(\mathrm{X})} P(\mathrm{X}_k,t_k)a_j^*(\mathrm{X}_k)(t_{k+1}-t_k).\end{aligned} \qquad (2.7)$$

If we multiple the memory chemical master equation (2.5) by all the states X at time t, sum over all these system states, and then re-index the summation on the right-hand side, we can obtain the equation for the mean $\overline{\mathrm{X}(t)}$, given by

$$\frac{d\overline{\mathrm{X}(t)}}{dt} = \sum_{j=1}^{m} \upsilon_j \overline{a_j(\mathrm{X}(t))} + \sum_{j=m+1}^{M} \upsilon_j \overline{a_j(\mathrm{X}(t))}\int_{t-\mu_j}^{t} \overline{a_j^*(\mathrm{X}(s))}ds + \sum_{j=m+1}^{M} \omega_j \overline{a_j^*(\mathrm{X}(t-\mu_j))} \qquad (2.8)$$

When all molecular numbers are very large and fluctuations in molecular numbers are not important, we can obtain the memory reaction rate equation, given by

$$\frac{d\mathrm{X}(t)}{dt} = \sum_{j=1}^{m} \upsilon_j a_j(\mathrm{X}(t)) + \sum_{j=m+1}^{M} \upsilon_j a_j(\mathrm{X}(t))\int_{t-\mu_j}^{t} a_j^*(\mathrm{X}(s))ds + \sum_{j=m+1}^{M} \omega_j a_j^*(\mathrm{X}(t-\mu_j)). \qquad (2.9)$$

The first term on the right-hand size of (2.9) describes the change of system state due to the firing of non-memory reactions; the second term is for the memory reactions whose firing is conditional to the firing of the trigger reaction during the memory time period $[t-\mu_j,t)$; and the final term is based on the transition of the memory species back to normal species. Note that the last term is different from the terms used in the delay reaction rate equation. For the trigger reaction R_j^* firing at $t-\mu_j$, the products have already produced when it fires. The last term in (2.9) represents an additional step for returning the memory species back to normal species.

The explicit Euler method for solving the above memory reaction rate equation is

$$\begin{aligned}\mathrm{X}(t_{n+1}) = \mathrm{X}(t_n) &+ h\sum_{j=1}^{m} \upsilon_j a_j(\mathrm{X}(t_n)) + h\sum_{j=m+1}^{M} \upsilon_j a_j(\mathrm{X}(t_n))\int_{t_n-\mu_j}^{t_n} a_j^*(\mathrm{X}(s))ds \\ &+ h\sum_{j=m+1}^{M} \omega_j a_j^*(\mathrm{X}(t_n-\mu_j))\end{aligned} \qquad (2.10)$$

Here $h = t_{n+1} - t_n$, and the integration in (2.10) is numerically calculated by

$$\int_{t_n-\mu_j}^{t_n} a_j^*(X(s))\,ds \approx \sum_{k=0}^{Nj-1} a_j^*(X(t_k))(t_{k+1} - t_k) \tag{2.11}$$

based on the partition $t - \mu_j = t_0 < t_1 < \ldots < t_{Nj} = t$ and the stepsize $(h_1 = t_{k+1} - t_k)$ satisfies $h_1 \le h$.

In the SSA setting Gillespie [5] introduced the concept of Poisson τ-leap methods in order to speed up the computational performance of the SSA. In this method a larger time step than that taken in the SSA is used and a number of reactions are allowed to fire within this step with a frequency drawn from a Poisson distribution. This method can also be used as a convergent technique to describe the evolution of molecular concentrations when noise is still important but the representation is continuous rather than discrete. We can adopt the same approach for the MSSA and this gives rise to the memory Poisson τ-leap method, given by

$$\begin{aligned} X(t_{n+1}) = X(t_n) &+ \sum_{j=1}^{m} \upsilon_j P\left[a_j(X(t_n))h\right] + \sum_{j=m+1}^{M} \upsilon_j P\left[a_j(X(t_n))h\int_{t_n-\mu_j}^{t_n} a_j^*(X(s))\,ds\right] \\ &+ \sum_{j=m+1}^{M} \omega_j P\left[a_j^*(X(t_n - \mu_j))h\right]. \end{aligned} \tag{2.12}$$

where $P(\lambda)$ is a Poisson random variable with mean λ. Note that the integral in (2.12) is calculated by using (2.11) numerically. If the mean of the Poisson random variable $P(\lambda)$ is large, it can be approximated by the Gaussian random variable $N(\lambda,\lambda) = \lambda + \sqrt{\lambda}N(0,1)$ whose mean and variance all are λ. Thus if the mean of the Poisson random variables in (2.12) is large, the Poisson scheme (2.12) can be approximated by

$$\begin{aligned} X(t_{n+1}) = X(t_n) &+ \sum_{j=1}^{m} \upsilon_j a_j(X(t_n))h + \sum_{j=1}^{m} \upsilon_j \sqrt{a_j(X(t_n))h}\, I_{nj1} \\ &+ \sum_{j=m+1}^{M} \upsilon_j a_j(X(t_n))h\int_{t_n-\mu_j}^{t_n} a_j^*(X(s))\,ds + \sum_{j=m+1}^{M} \upsilon_j \sqrt{a_j(X(t_n))h\int_{t_n-\mu_j}^{t_n} a_j^*(X(s))\,ds}\, I_{nj2} \\ &+ \sum_{j=m+1}^{M} \omega_j a_j^*(X(t_n - \mu_j))h + \sum_{j=m+1}^{M} \omega_j \sqrt{a_j^*(X(t_n - \mu_j))h}\, I_{nj3}. \end{aligned} \tag{2.13}$$

where $I_{njk} \sim N(0,1)$ is the standard Gaussian random variable. When $h \to 0$, this gives rise to a system of stochastic memory differential equations that describe the evolution of chemical concentrations for chemical systems with memory reactions, given by

$$\begin{aligned} dX = &\sum_{j=1}^{m} \upsilon_j a_j(X(t))dt + \sum_{j=1}^{m} \upsilon_j \sqrt{a_j(X(t))}\,dW_{nj1}(t) \\ &+ \sum_{j=m+1}^{M} \left[\upsilon_j a_j(X(t))\int_{t-\mu_j}^{t} a_j^*(X(s))\,ds\right]dt + \sum_{j=m+1}^{M} \upsilon_j \sqrt{a_j(X(t))\int_{t-\mu_j}^{t} a_j^*(X(s))\,ds}\,dW_{nj2}(t) \\ &+ \sum_{j=m+1}^{M} \omega_j a_j^*(X(t - \mu_j))dt + \sum_{j=m+1}^{M} \omega_j \sqrt{a_j^*(X(t - \mu_j))}\,dW_{nj3}(t). \end{aligned} \tag{2.14}$$

where $W_{njk}(t)$ is the Wiener process whose increment $\Delta W_{njk}(t) = W_{njk}(t+\Delta) - W_{njk}(t) \sim N(0,\Delta)$ is a Gaussian random variable.

2.2 Memory master chemical equations for systems with time delay

Here we consider a biological system with $M_1 (= m_1)$ non-memory reactions $(R_1,\ldots,R_{M_1})$; m_2 non-memory delayed reactions $(R_{M_1+1},\ldots,R_{M_2})$, where $M_2 = m_1 + m_2$; m_3 memory reactions $(R_{M_2+1},\ldots,R_{M_3})$, where $M_3 = m_1 + m_2 + m_3$, and m_4 delayed memory reactions $(R_{M_3+1},\ldots,R_{M_4})$, where $M_4 = m_1 + m_2 + m_3 + m_4$. For each memory reaction $R_j, j \in \{M_3+1,\ldots,M_4\}$, there is a corresponding trigger reaction R_j^* whose propensity function is denoted as $a_j^*(\mathrm{X})$. Since the trigger reaction is a non-memory reaction, thus $a_j^*(\mathrm{X})$ is one of the propensity functions $a_i(\mathrm{X})$ $(i \in \{1,\ldots,M_2\})$. Note that a trigger reaction starts a memory time period that may include a number of memory reactions.

Here we are interested in the probability function of the system state $\mathrm{X}(t)$ at time t. We will derive the time evolution equation of the probability function

$$P(\mathrm{X},t) \equiv \mathrm{Prob}\{\mathrm{X}(t) = \mathrm{X} \mid \mathrm{X}(t_0) = \mathrm{X}_0, \text{ and } \mathrm{X}(t) = \Phi(t), t \le t_0\}, \qquad (2.15)$$

where $\Phi(t)$ is the function for the initial condition of delayed reactions. We take a time increment dt which is so small that the probability for two or more reactions to occur in dt is negligible compared to the probability for at most one reaction. It is assumed that the reaction time of an elementary reaction or the consuming part of a delayed reaction is dt. For a reaction with time delay τ_j firing at time t, the product will manifest at time $t + \tau_j + dt$.

For a chemical system with both delayed reactions and memory reactions, the master equation is based on the current system state at time t for elementary reactions, the system state at $t - \tau_j$ for a delayed reaction with time delay τ_j, the system states during the interval $[t - \mu_j, t]$ for a memory reaction whose memory time period has length μ_j, and the system states during the interval $[t - \tau_j - \mu_j, t - \tau_j]$ for a delayed memory reaction with delay τ_j and memory interval length μ_j. Then the probability of the system being in the state X at $t + dt$ is given by

$$
\begin{aligned}
P(X,t+dt) = {} & P(X,t) - \sum_{j=1}^{M_1} a_j(X)P(X,t)dt + \sum_{j=1}^{M_1} a_j(X-v_j)P(X-v_j,t)dt \\
& - \sum_{j=M_1+1}^{M_2} \sum_{X_i \in I(X)} a_j(X_i)P(X,t;X_i,t-\tau_j)dt + \sum_{j=M_1+1}^{M_2} \sum_{X_i \in I(X)} a_j(X_i)P(X-v_j,t;X_i,t-\tau_j)dt \\
& - \sum_{j=M_2+1}^{M_3} \sum_{k=0}^{Nj-1} \sum_{X_l \in I(X)} a_j(X)dtP(X,t;X_l,t_k)a_j^*(X_l)(t_{k+1}-t_k) \\
& + \sum_{j=M_2+1}^{M_3} \sum_{k=0}^{Nj-1} \sum_{X_l \in I(X)} a_j(X-v_j)dtP(X-v_j,t;X_l,t_k)a_j^*(X_l)(t_{k+1}-t_k) \qquad (2.16) \\
& - \sum_{j=M_3+1}^{M_4} \sum_{k=0}^{Nj-1} \sum_{X_l \in I(X)} \sum_{X_i \in I(X)} a_j(X_i)dtP(X,t;X_i,t-\tau_j;X_l,t_k)a_j^*(X_l)(t_{k+1}-t_k) \\
& + \sum_{j=M_3+1}^{M_4} \sum_{k=0}^{Nj-1} \sum_{X_l \in I(X)} \sum_{X_i \in I(X)} a_j(X_i)dtP(X-v_j,t;X_i,t-\tau_j;X_l,t_k)a_j^*(X_l)(t_{k+1}-t_k) \\
& - \sum_{j=M_2+1}^{M_4} \sum_{X_l \in I(X)} a_j^*(X_l)P(X,t;X_l,t-\mu_j)dt + \sum_{j=M_2+1}^{M_4} \sum_{X_l \in I(X)} a_j^*(X_l)P(X-\omega_j,t;X_l,t-\mu_j)dt
\end{aligned}
$$

where $P(X,t;X_i,t-\tau_j;X_k,t_k)$ is the probability that the system is in the state X at time t, in the state X_i at time $t-\tau_j$, and in the state X_k at t_k. In addition, $I(X)$ is the set of all possible system states, and $t-\mu_j = t_0 < t_1 < \ldots < t_{Nj} = t$ is a partition of the interval $[t-\mu_j,t]$ with stepsize $dt = t_{k+1} - t_k$. Note that in the last two items in (2.16), we used the same notation to represent the partition $t-\tau_j-\mu_j = t_0 < t_1 < \ldots < t_{Nj} = t-\tau_j$ of the interval $[t-\tau_j-\mu_j, t-\tau_j]$.

The first part of equation (2.16) (the 2nd~5th items) describes the dynamics of both the elementary non-memory reactions and delayed non-memory reactions, which is the same as the delay chemical master equation [6]. The second part (the 6th~7th items) describes the change of system state due to the firing of elementary memory reactions. The 8th~9th items represent the change of system states due to the firing of the delayed memory reactions at time $t-\tau_j$ and these reactions depend on the trigger reaction firing during the time period $[t-\tau_j-\mu_j, t-\tau_j]$. The last two terms in (2.16) represent the transfer of memory species back to non-memory species when the memory time period (for both non-delayed and delayed memory reactions) finishes. Note that the finish of a memory time period is independent of the manifest of the delayed memory reactions that fire during the memory time period.

If the length μ_j of the memory time period is large and there are relatively large number of reactions firing in the time interval $[t-\mu_j,t)$, it was assumed that the coupling of the system states at t and $t_k \in [t-\mu_j,t)$ is weak, namely the firing of the memory reaction R_j is independent of the trigger reaction R_j^*. Then we have

$$P(\mathrm{X},t;\mathrm{X}_l,t_k) \approx P(\mathrm{X},t)P(\mathrm{X}_l,t_k). \tag{2.17}$$

In addition, $a_j(\mathrm{X}_i)P(\mathrm{X},t;\mathrm{X}_i,t-\tau_j)$ represents the probability that one delayed reaction R_j (with delay τ_j) fires at $t-\tau_j$, when the system states are X and X_i at time t and $t-\tau_j$, respectively. If the time delays are large and there is a relatively large number of reactions in the time interval $[t-\tau_j,t)$, it was assumed that the coupling of the system states at t and $t-\tau_j$ is weak, and it gives [6]

$$P(\mathrm{X},t;\mathrm{X}_i,t-\tau_j) \approx P(\mathrm{X},t)P(\mathrm{X}_i,t-\tau_j) \tag{2.18}$$

The assumptions (2.17) and (2.18) lead to

$$P(\mathrm{X},t;\mathrm{X}_i,t-\tau_j;\mathrm{X}_l,t_k) \approx P(\mathrm{X},t)P(\mathrm{X}_i,t-\tau_j)P(\mathrm{X}_l,t_k) \tag{2.19}$$

which represents the coupling of the system states X at t, X_i at $t-\tau_j$ and X_l at $t_k \in [t-\mu_j-\tau_j, t-\tau_j]$ is weak.

When $dt \to 0$, this lead to the memory chemical master equation

$$\begin{aligned}
\frac{\partial}{\partial t}P(\mathrm{X},t) = &-\sum_{j=1}^{M_1} a_j(\mathrm{X})P(\mathrm{X},t) + \sum_{j=1}^{M_1} a_j(\mathrm{X}-\upsilon_j)P(\mathrm{X}-\upsilon_j,t) \\
&- \sum_{j=M_1+1}^{M_2} \overline{a_j(\mathrm{X}(t-\tau_j))}P(\mathrm{X},t) + \sum_{j=M_1+1}^{M_2} \overline{a_j(\mathrm{X}(t-\tau_j))}P(\mathrm{X}-\upsilon_j,t) \\
&- \sum_{j=M_2+1}^{M_3} a_j(\mathrm{X})P(\mathrm{X},t)\int_{t-\mu_j}^{t} \overline{a_j^*(\mathrm{X}(s))}\,ds + \sum_{j=M_2+1}^{M_3} a_j(\mathrm{X}-\upsilon_j)P(\mathrm{X}-\upsilon_j,t)\int_{t-\mu_j}^{t} \overline{a_j^*(\mathrm{X}(s))}\,ds \\
&- \sum_{j=M_3+1}^{M_4} P(\mathrm{X},t)\overline{a_j(\mathrm{X}(t-\tau_j))}\int_{t-\tau_j-\mu_j}^{t-\tau_j} \overline{a_j^*(\mathrm{X}(s))}\,ds \\
&+ \sum_{j=M_3+1}^{M_4} P(\mathrm{X}-\upsilon_j,t)\overline{a_j(\mathrm{X}(t-\tau_j))}\int_{t-\tau_j-\mu_j}^{t-\tau_j} \overline{a_j^*(\mathrm{X}(s))}\,ds \\
&- \sum_{j=M_3+1}^{M_4} \overline{a_j^*(\mathrm{X}(t-\mu_j))}P(\mathrm{X},t) + \sum_{j=M_3+1}^{M_4} \overline{a_j^*(\mathrm{X}(t-\mu_j))}P(\mathrm{X}-\omega_j,t),
\end{aligned} \tag{2.20}$$

where the mean of the propensity functions of the delayed reactions is derived from

$$\overline{a_j(X(t-\tau_j))} = \sum_{X_i \in I(X)} a_j(X_i)P(X_i,t-\tau_j),$$

in addition, the mean of the propensity functions of trigger reactions $a_j^*(X(t))$ and its integration are derived from (2.6) and (2.7), respectively. If we multiple the memory chemical master equation (2.20) by all of the states X at time t, sum over all these system states, and then re-index the summation on the right-hand side, we can obtain the equation for the mean $\overline{\mathrm{X}(t)}$, given by

$$\frac{d\overline{X(t)}}{dt} = \sum_{j=1}^{M_1} \upsilon_j \overline{a_j(X(t))} + \sum_{j=M_1+1}^{M_2} \upsilon_j \overline{a_j(X(t-\tau_j))} + \sum_{j=M_2+1}^{M_3} \upsilon_j \overline{a_j(X(t))} \int_{t-\mu_j}^{t} \overline{a_j^*(X(s))}\, ds \\ + \sum_{j=M_3+1}^{M_4} \upsilon_j \overline{a_j(X(t-\tau_j))} \int_{t-\tau_j-\mu_j}^{t-\tau_j} \overline{a_j^*(X(s))}\, ds + \sum_{j=M_2+1}^{M_4} \omega_j \overline{a_j^*(X(t-\mu_j))}. \tag{2.21}$$

When all molecular numbers are very large and fluctuations are not important, we can get the memory reaction rate equation, given by

$$\frac{dX(t)}{dt} = \sum_{j=1}^{M_1} \upsilon_j a_j(X(t)) + \sum_{j=M_1+1}^{M_2} \upsilon_j a_j(X(t-\tau_j)) + \sum_{j=M_2+1}^{M_3} \upsilon_j a_j(X(t)) \int_{t-\mu_j}^{t} a_j^*(X(s))\, ds \\ + \sum_{j=M_3+1}^{M_4} \upsilon_j a_j(X(t-\tau_j)) \int_{t-\tau_j-\mu_j}^{t-\tau_j} a_j^*(X(s))\, ds + \sum_{j=M_2+1}^{M_4} \omega_j a_j^*(X(t-\mu_j)). \tag{2.22}$$

The explicit Euler method for solving the above memory reaction rate equation is

$$X(t_{n+1}) = X(t_n) + h\sum_{j=1}^{M_1} \upsilon_j a_j(X(t_n)) + h \sum_{j=M_1+1}^{M_2} \upsilon_j a_j(X(t_n-\tau_j)) \\ + h \sum_{j=M_2+1}^{M_3} \upsilon_j a_j(X(t_n)) \int_{t_n-\mu_j}^{t_n} a_j^*(X(s))\, ds + h \sum_{j=M_2+1}^{M_4} \omega_j a_j^*(X(t_n-\mu_j)) \\ + h \sum_{j=M_3+1}^{M_4} \upsilon_j a_j(X(t_n-\tau_j)) \int_{t-\tau_j-\mu_j}^{t-\tau_j} a_j^*(X(s))\, ds. \tag{2.23}$$

Here $h = t_{n+1} - t_n$. The integration in (2.23) is numerically calculated by (2.11) based on the partition $t-\mu_j = t_0 < t_1 < \ldots < t_{NK} = t$ or $t-\tau_j-\mu_j = t_0 < t_1 < \ldots < t_{NK} = t-\tau_j$, and the stepsize $(h_1 = t_{k+1} - t_k)$ satisfies $h_1 \le h$.

If we adopt the tau-leap approach [5] for the memory-SSA, this gives rise to the memory Poisson τ-leap method, given by

$$X(t_{n+1}) = X(t_n) + \sum_{j=1}^{M_1} \upsilon_j P[a_j(X(t_n))h] + \sum_{j=M_1+1}^{M_2} \upsilon_j P[a_j(X(t_n-\tau_j))h] \\ + \sum_{j=M_2+1}^{M_3} \upsilon_j P\left[a_j(X(t_n))h \int_{t_n-\mu_j}^{t_n} a_j^*(X(s))\, ds\right] + \sum_{j=M_2+1}^{M_4} \omega_j P\left[a_j^*(X(t_n-\mu_j))h\right] \\ + \sum_{j=M_3+1}^{M_4} \upsilon_j P\left[a_j(X(t_n-\tau_j))h \int_{t-\tau_j-\mu_j}^{t-\tau_j} a_j^*(X(s))\, ds\right], \tag{2.24}$$

where $P(\lambda)$ is a Poisson random variable with mean λ. If the mean λ is large, the Poisson random variable can be approximated by a Gaussian random variable, namely $P(\lambda) \approx N(\lambda, \lambda)$. When the mean of the Poisson random variables in (2.24) is large, the Poisson τ-leap scheme (2.24) can be represented by

$$\begin{aligned}
X(t_{n+1}) = X(t_n) &+ \sum_{j=1}^{M_1} \upsilon_j a_j(X(t_n))h + \sum_{j=1}^{M_1} \upsilon_j \sqrt{a_j(X(t_n))h} I_{nj1} \\
&+ \sum_{j=M_1+1}^{M_2} \upsilon_j a_j(X(t_n-\tau_j))h + \sum_{j=M_1+1}^{M_2} \upsilon_j \sqrt{a_j(X(t_n-\tau_j))h} I_{nj2} \\
&+ \sum_{j=M_2+1}^{M_3} \upsilon_j a_j(X(t_n))h \int_{t_n-\mu_j}^{t_n} a_j^*(X(s))ds + \sum_{j=M_2+1}^{M_3} \upsilon_j \sqrt{a_j(X(t_n))h \int_{t_n-\mu_j}^{t_n} a_j^*(X(s))ds} I_{nj3} \\
&+ \sum_{j=M_2+1}^{M_4} \omega_j a_j^*(X(t_n-\mu_j))h + \sum_{j=M_3+1}^{M_4} \upsilon_j a_j(X(t_n-\tau_j))h \int_{t_n-\tau_j-\mu_j}^{t_n-\tau_j} a_j^*(X(s))ds \\
&+ \sum_{j=M_2+1}^{M_4} \omega_j \sqrt{a_j^*(X(t_n-\mu_j))h} I_{nj4} + \sum_{j=M_3+1}^{M_4} \upsilon_j \sqrt{a_j(X(t_n-\tau_j))h \int_{t_n-\tau_j-\mu_j}^{t_n-\tau_j} a_j^*(X(s))ds} I_{nj5},
\end{aligned} \tag{2.25}$$

where $I_{nji} \sim N(0,1)\,(i = 1 \sim 5)$. When $h \to 0$, this gives rise to systems of stochastic memory differential equations that describe the evolution of chemical concentrations for chemical systems with memory reactions, given by

$$\begin{aligned}
dX = &\sum_{j=1}^{M_1} \upsilon_j a_j(X(t))dt + \sum_{j=1}^{M_1} \upsilon_j \sqrt{a_j(X(t))} dW_{j1}(t) \\
&+ \sum_{j=M_1+1}^{M_2} \upsilon_j a_j(X(t-\tau_j))dt + \sum_{j=M_1+1}^{M_2} \upsilon_j \sqrt{a_j(X(t-\tau_j))} dW_{j2}(t) \\
&+ \sum_{j=M_2+1}^{M_3} \left[\upsilon_j a_j(X(t)) \int_{t-\mu_j}^{t} a_j^*(X(s))ds\right]dt + \sum_{j=M_2+1}^{M_3} \upsilon_j \sqrt{a_j(X(t)) \int_{t-\mu_j}^{t} a_j^*(X(s))} dW_{j3}(t) \\
&+ \sum_{j=M_2+1}^{M_4} \omega_j a_j^*(X(t-\mu_j))dt + \sum_{j=M_3+1}^{M_4} \left[\upsilon_j a_j(X(t-\tau_j)) \int_{t-\tau_j-\mu_j}^{t-\tau_j} a_j^*(X(s))ds\right]dt \\
&+ \sum_{j=M_2+1}^{M_4} \omega_j \sqrt{a_j^*(X(t-\mu_j))} dW_{j4}(t) + \sum_{j=M_3+1}^{M_4} \upsilon_j \sqrt{a_j(X(t-\tau_j)) \int_{t-\tau_j-\mu_j}^{t-\tau_j} a_j^*(X(s))ds} dW_{j5}(t),
\end{aligned} \tag{2.26}$$

where $W_{njk}(t)$ is the Wiener process whose increment $\Delta W_{njk}(t) = W_{njk}(t+\Delta) - W_{njk}(t) \sim N(0,\Delta)$ is a Gaussian random variable.

When the chemical reaction system includes elementary reactions and delayed reactions only, the memory reaction rate equation (2.22) is the delayed reaction rate equation [6]

$$\frac{dX(t)}{dt} = \sum_{j=1}^{M_1} \upsilon_j a_j(X(t)) + \sum_{j=M_1+1}^{M_2} \upsilon_j a_j(X(t-\tau_j)) \tag{2.27}$$

and the stochastic memory differential equation (2.26) is the delayed stochastic differential equation [6], given by

$$\begin{aligned}
dX = &\sum_{j=1}^{M_1} \upsilon_j a_j(X(t))dt + \sum_{j=1}^{M_1} \upsilon_j \sqrt{a_j(X(t))} dW_{j1}(t) \\
&+ \sum_{j=M_1+1}^{M_2} \upsilon_j a_j(X(t-\tau_j))dt + \sum_{j=M_1+1}^{M_2} \upsilon_j \sqrt{a_j(X(t-\tau_j))h} dW_{j2}(t).
\end{aligned} \tag{2.28}$$

Comparing with the delayed reaction rate equation (2.27), the transfer of memory species (the 4th term on the right hand side of (2.10)) is similar to the delayed reactions in (2.27). However, the transferring event in (2.10) is additional to the firing of memory reaction (the 3rd term on the right hand side of (2.10)) and it is not associated with the stoichiometrics of the trigger reaction. In addition, the elementary memory reactions in (2.10) are instant events and their firing are conditional to the firing of the memory trigger reaction during the past time period.

3. A stochastic model with memory for single-gene expression

3.1 Model assumptions

A stochastic model with memory reactions was proposed to realize the bursting expression of a single gene. This stochastic model is based on the following assumptions.

1. This network contains a single gene. It was assumed that there is no auto feedback loop from the gene product to gene transcription.
2. The DNA is activated by the binding of a transcription factor (TF) to a specific response element in the promoter region (reaction 3.1); and then the TF acts as a platform to recruit RNAP to the local promoter region to form the pre-initiation complex (3.2), from which transcription can start.
3. The first transcription (3.3) is the trigger reaction of the first memory time period for gene transcription. This reaction transfers the complex DNA-TF to the memory species M(DNA-TF). This memory species was assumed to bind together during the memory time period of gene transcription.
4. IS(mRNA) in (3.3) represents the imaginary species of mRNA during the delayed time period of transcription elongation. When the reaction product manifests, this imaginary species is updated to mRNA via reaction (3.14).
5. Once the successful pre-initiation complex has been formed in (3.3), re-initiation occurs with much higher probability in reaction (3.4) [7]. The activated transcription starting site allows for the competitive binding of a number of initiating complexes, which is represented by RNAP, and multiple initiation events occur during one transcription cycle via reaction (3.4) and (3.5) [8].
6. The binding of deactivating complex turns gene off at the end of the transcription memory period. The long time period of gene inactivation was realized by the second memory time period that immediately follows the memory time period of gene transcription. The memory species in the gene inactivation window is M(DNA). It was assumed that M(DNA) could recruit IF only via reaction (3.6), but M(DNA)-TF could not recruit RNAP. Thus the lack of the pre-

initiation complex excludes the possibility of gene expression during the second memory time period.

7. It was assumed that the length of each memory time period is either a constant λ_i $(i = 1,2)$ or an exponential random variable $P(\lambda_i)$ with mean λ_i.
8. It was assumed that the lengths of the transcription memory window and gene inactivation window were $\lambda_1 = 10$ min and $\lambda_2 = 50$ min, respectively [8].
9. The time delay in gene transcription was assumed to be 33 min that includes the time of elongation, splicing and mRNA translocation [9].
10. Translation was modelled as a first-order reaction (3.6) and the time delay in translation was not considered in this model.
11. The degradation of mRNA and protein was modelled as the first-order reactions (3.7) and (3.8), respectively.
12. According to the possible states of memory species M(DNA-TF), namely either M(DNA-TF) and M(DNA-TF)-RNAP, two transfer reactions (2.9 and 2.10) were defined for transferring M(DNA-TF) into the second memory species M(DNA).
13. According to the possible states of memory species M(DNA), , namely M(DNA) and M(DNA)-TF, two transfer reactions (2.11 and 2.12) were needed to transfer memory species M(DNA) back to the non-memory species DNA.

3.2 Chemical reactions

Chemical reactions in the proposed stochastic model are listed below

$$\text{TF + DNA} \underset{d1}{\overset{a1}{\rightleftarrows}} \text{DNA-TF} \tag{3.1}$$

$$\text{DNA-TF + RNAP} \underset{d2}{\overset{a2}{\rightleftarrows}} \text{DNA-TF-RNAP} \tag{3.2}$$

$$\text{DNA-TF-RNAP} \xrightarrow{k1} \text{M(DNA-TF) + RNAP + IS(mRNA)} \tag{3.3}$$

$$\text{M(DNA-TF)+RNAP} \underset{d3}{\overset{a3}{\rightleftarrows}} \text{M(DNA-TF)-RNAP} \tag{3.4}$$

$$\text{M(DNA-TF)-RNAP} \xrightarrow{k2} \text{M(DNA-TF)+RNAP + IS(mRNA)} \tag{3.5}$$

$$\text{TF + M(DNA)} \underset{d4}{\overset{a4}{\rightleftarrows}} \text{M(DNA)-TF} \tag{3.6}$$

$$\text{mRNA} \xrightarrow{k3} \text{mRNA + P} \tag{3.7}$$

$$\text{mRNA} \xrightarrow{k4} () \tag{3.8}$$

$$P \xrightarrow{k5} () \quad (3.9)$$

Reactions for transferring M(DNA-TF) to M(DNA)

M(DNA-TF-RNAP) → M(DNA) + TF + RNAP + IS(mRNA), (3.10)

M(DNA-TF) → M(DNA) + TF. (3.11)

Reactions for transferring M(DNA) back to DNA

M(DNA) → DNA (3.12)

M(DNA)-TF → DNA + TF (3.13)

Reaction for the manifest of delayed reaction (2.3) or (2.5)

IS(mRNA) → mRNA (3.14)

3.3 Initial conditions

Initial conditions regarding the molecular numbers are [DNA]=1, [TF]=2000, [RNAP]=2000. All other variables are zero.

3.4 Rate constants

STable 1: Rate constants of the stochastic model of a single gene.

Rate	Value	Comment
a_1	0.0001	[8]
d_1	0.01	[8]
a_2	0.21	[7]
d_2	0.05	[7]
k_1	0.26	[7]
a_3	0.63	The transcription reinitiation is 3 folds faster than gene initiation [7]
d_3	0.05	The same as d_2
k_2	0.78	The same comment as a_3
a_4	0.0001	The same value as a_1
d_4	0.01	The same value as d_1
k_3	1	[10]
k_4	0.0231	The half-life of mRNA is assumed to be 30 min.
k_5	0.0116	The half-life of protein is assumed to be 60 min.

4. Stochastic model with memory reactions for the p53-MDM2 core module

4.1 Model assumptions

The proposed stochastic model with memory reactions for the p53 gene network is based on the following assumptions.

1. The regulatory network includes two genes: p53 and MDM2.

2. Following the assumptions in the published models [9, 11, 12], it was assumed that p53 is transcribed constitutively (reaction 4.3).
3. The half-life of p53 without interacting with MDM2 is as long as 7.3 hours (reaction 4.4) [13]. MDM2 negatively regulated p53 activity by promoting its ubiquitination (reaction 4.5). The ubiquitinated p53 proteins, which is denoted as p53-ubi, turnover quickly with half-life about 30 min (4.6) [14].
4. Under various stress conditions, p53 is activated by phosphorylation through upstream mediators including kinase ATM and Chk2 [15]. The activated p53 is denoted as p53*. The activities of kinases ATM in Figure 5A of Ref [15] was used to represented the upstream signal activities, which was realized in the ATM dependent p53 protein phosphorylation rate in reaction (4.7)

$$k_5 = k_{50}[ATM]\frac{[53]}{K_5 + [p53]} \qquad (4.1)$$

where k_{50} is the maximal phosphorylation rate of p53 proteins.
5. The activated p53* can be dephosphorylated by a number of phosphatase [16], and we realized the dephosphorylation by a first order reaction (4.8).
6. The posttranslational modification of p53 disrupts the p53-MDM2 interaction, though MDM2 can still regulate the activated p53* by promoting its ubiquitination and degradation. The association rate of MDM2 with p53* in reaction (4.9) is 10 folds less than that of MDM2 with p53 in (4.3) [9]. In addition, the phosphorylation can extend the half-life of p53* proteins significantly to about 210 min [14] [17] in reaction (4.10).
7. The ubiquitinated p53 proteins can be deubiquitinated by HUASP [18], and it was assumed that the deubiquitination of p53 proteins was the first-order reactions (4.11) and (4.12).
8. Gene MDM2 has two promoters. One is constitutively active and the other is activated by p53 [19]. It was assumed that the basal transcription maintain the MDM2 copy number at the basal level (4.11). This basal level was assumed to be the copy number measured before the activation of p53 [20].
9. The half-life of protein Mdm2 in the unstressed cell is about 30 min while that in DNA-damaged cells is as short as 5 min [21, 22]. It was assumed that the probability of MDM2 degradation in (4.12) also depends on the activity of the upstream regulator and follows the Michaelis-Menten mechanism.

$$k_{10} = k_{101}[MDM2] + k_{102}\frac{[MDM2][ATM]}{K_{10} + [MDM2]} \qquad (4.2)$$

The constant k_{101} and K_{102} were estimated by simulation to match the measured MDM2 numbers in [20].

10. The activated p53* proteins form homogeneous dimers (4.13), and the p53 tetramer is assembled as a "dimer of dimer" (4.14) [23]. All forms of p53* can bind to the promoter site of MDM2 gene. However, the binding of p53 monomer or dimer to gene MDM2 is transient and unstable. Only p53 tetramer can form a stable complex with MDM2 gene [24]. Thus it was assumed that the p53 tetramer is the TF of gene MDM2 (4.15).
11. RNAP proteins bind to gene MDM2 binding site in the promoter region (4.16 and 4.17) and start transcription initiation. It was assumed that the transcription of gene MDM2 is a memory process. The trigger reaction (4.16) transfers the complex p53*4-DNA into the memory species M(p53*4-DNA). According to the experimentally measured MDM2 copy numbers [20], it was estimated that the length of the transcription memory time period is 1.0 hour. The memory species was assumed to bind together during the memory time period.
12. Following the data of gene initiation rate [7], it was assumed that transcription re-initiation rate of gene MDM2 in (4.17) was 3 times faster than that of transcription initiation in (4.14).
13. The time delay in gene transcription was assumed to be 33 min that includes the time of elongation, splicing and mRNA translocation [9]. The manifest of the delayed reaction is realized by (4.26).
14. When gene DMD2 turns off, it was assumed that the second memory time period for gene inactivation immediately followed the memory time period of gene transcription. Memory species M(p53*4-DNA) in the first time period is transferred to the memory species M(DNA) via reactions (4.22, 4.23).
15. According to the possible states of memory species M(DNA), two transfer reactions (4.24, 4.25) were designed to transfer memory species M(DNA) back to normal species DNA.

4.2 Biochemical reactions

$$() \xrightarrow{k_1} \text{p53}, \tag{4.3}$$

$$\text{p53} \xrightarrow{k_2} () \tag{4.4}$$

$$\text{p53} + \text{MDM2} \underset{d_3}{\overset{a_3}{\rightleftharpoons}} \text{p53-MDM2} \xrightarrow{k_3} \text{MDM2} + \text{p53-ubi} \tag{4.5}$$

$$\text{p53-ubi} \xrightarrow{k_4} () \tag{4.6}$$

$$\text{p53} \xrightarrow{k_5} \text{p53*} \tag{4.7}$$

$$\text{p53*} \xrightarrow{k_6} \text{p53} \tag{4.8}$$

$$\text{p53* + MDM2} \underset{d_7}{\overset{a_7}{\rightleftarrows}} \text{p53*-MDM2} \xrightarrow{k_7} \text{MDM2 + p53*-ubi} \quad (4.9)$$

$$\text{p53*-ubi} \xrightarrow{k_8} () \quad (4.10)$$

$$\text{p53-ubi} \xrightarrow{k_{18}} \text{p53} \quad (4.11)$$

$$\text{p53*-ubi} \xrightarrow{k_{19}} \text{p53*} \quad (4.12)$$

$$() \xrightarrow{k_9} \text{MDM2, The basal transcription} \quad (4.11)$$

$$\text{MDM2} \xrightarrow{k_{10}} () \quad (4.12)$$

$$\text{p53* + p53*} \underset{d_{12}}{\overset{a_{11}}{\rightleftarrows}} \text{p53*2} \quad (4.13)$$

$$\text{p53*2 + p53*2} \underset{d_{12}}{\overset{a_{12}}{\rightleftarrows}} \text{p53*4 (About 15\% of total p53 is in the tetramer)} \quad (4.14)$$

$$\text{p53*4 + DNA} \underset{d_{13}}{\overset{a_{13}}{\rightleftarrows}} \text{p53*4- DNA} \quad (4.15)$$

$$\text{p53*4- DNA + RNAP} \underset{d_{14}}{\overset{a_{14}}{\rightleftarrows}} \text{p53*4- DNA-RNAP} \quad (4.16)$$

$$\xrightarrow{k_{14}} \text{M(p53*4- DNA) + RNAP + IS(mRNA)}$$

$$\text{M(p53*4- DNA) + RNAP} \underset{d_{15}}{\overset{a_{15}}{\rightleftarrows}} \text{M(p53*4- DNA)-RNAP} \quad (4.17)$$

$$\xrightarrow{k_{15}} \text{M(p53*4- DNA)-R + RNAP + IS(mRNA)}$$

$$\text{mRNA} \xrightarrow{k_{16}} \text{mRNA + MDM2} \quad (4.18)$$

$$\text{mRNA} \xrightarrow{k_{17}} () \quad (4.19)$$

Transition of memory species of the first memory reaction

M(p53*4-DNA) → p53*4 + M(DNA) (4.22)

M(p53*4-DNA)-RNAP → p53*4 + M(DNA) + RNAP (4.23)

Transition of memory species of the first memory reaction

M(DNA) → DNA (4.24)

p53*4-M(DNA) → p53*-4 + M(DNA) (4.25)

Generating reactions of the delayed reactions

$$IS(mRNA) \rightarrow mRNA \quad (4.26)$$

4.3 Initial conditions

The initial molecule copy numbers are [p53]=14,000, [MDM2]=70,000 [20], [DNA]=1, [RNAP]=200. The initial copy numbers of other species are zero.

4.4 Kinetic Rate constant (unit: 1/min)

STable 2: Rate constants of the p53-MDM2 network

Rate	value	Comment
k_1	103.44	P53 basal transcription rate, estimated by simulation in order to maintain the basal p53 copy number (=14,000).
k_2	0.0016	P53 degradation rate of untacted p53, the half-life is ~7 hours [13].
a_3	0.1747	Association rate of p53 with MDM2 [25].
d_3	123.6	Disassociation rate of p53-MDM2 hetero-dimer [25].
k_3	4.8	Ubiquitin rate of p53 [26].
k_4	0.0231	Ubiquitined p53 degradation rate (~30 min) [14].
k_{50}	3×10^7	P53 phosphorylation rate, estimated by simulation to match the experimentally measured p53 copy numbers.
K_5	14,000	Constant in rate k_5 (4.1), assumed as the basal p53 number.
k_6	0.2	P53 deactivation rate [9].
a_7	0.01747	Association rate of p53 with MDM2, which is 10 folds less than a_3 [9].
d_7	123.6	The same as d_3.
k_7	4.8	The same as k_3.
k_8	0.0046	The half-life of the activated p53* is ~150min [27].
k_9	757.84	The basal expression rate of MDM2 is estimated by simulation to maintain the basal MDM2 copy number (=70,000).
k_{101}	0.0231	The half-life of protein Mdm2 in the unstressed cell is ~30 min [21].
k_{102}	1500	ATM-dependent degradation rate of MDM2, estimation by simulation matching the experimentally measured MDM2 copy numbers.
K_{10}	70,000	Constant in rate k_{10} (4.2), assumed as the basal MDM2 number.
a_{11}	0.02	P53 dimer formation rate [28]
d_{11}	5	Disassociation rate of p53 dimers [28]
a_{12}	0.02	P53 tetramer formation rate, the same as a_{11}
d_{12}	5	The same as d_{11}
a_{13}	4.089×10^{-5}	P53 tetramer association rate to the binding site of gene MDM2 [29]
d_{13}	0.0276	The half-life of p53 tetramer binding is ~25 min [24]
a_{14}	0.21	RNAP binding rate [7]
d_{14}	0.05	RNAP disassociation rate [7]

k_{14}	0.26	Transcription initiation rate [Ref 7].
a_{15}	0.063	Transcription re-initiation is 3 times faster than initiation rate a_{14} [7]
d_{15}	0.05	The same as d_{14}
k_{15}	0.78	The same comment as that for a_{15} [7]
k_{16}	20	MDM2 translational rate, estimated by simulation to match experimentally measured MDM2 protein numbers.
k_{17}	0.0058	The half-life of MDM2 mRNA is 60~120 min [30].
k_{18}	2.45	Deubiquitination rate of p53 [31].
k_{19}	2.45	Deubiquitination rate of p53 ($=k_{18}$)

References

1. Gillespie, D.T., *Exact Stochastic Simulation of Coupled Chemical-Reactions.* Journal of Physical Chemistry, 1977. **81**(25): p. 2340-2361.
2. Bratsun, D., et al., *Delay-induced stochastic oscillations in gene regulation.* Proc Natl Acad Sci U S A, 2005. **102**(41): p. 14593-8.
3. Barrio, M., et al., *Oscillatory regulation of Hes1: Discrete stochastic delay modelling and simulation.* PLoS Comput Biol, 2006. **2**(9): p. e117.
4. Choi, P.J., et al., *A stochastic single-molecule event triggers phenotype switching of a bacterial cell.* Science, 2008. **322**(5900): p. 442-6.
5. Gillespie, D.T., *Approximate accelerated stochastic simulation of chemically reacting systems.* Journal of Chemical Physics, 2001. **115**(4): p. 1716-1733.
6. Tian, T.H., et al., *Stochastic delay differential equations for genetic regulatory networks.* Journal of Computational and Applied Mathematics, 2007. **205**(2): p. 696-707.
7. Narayan, S., et al., *RNA polymerase II transcription. Rate of promoter clearance is enhanced by a purified activating transcription factor/cAMP response element-binding protein.* J Biol Chem, 1994. **269**(17): p. 12755-63.
8. Degenhardt, T., et al., *Population-level transcription cycles derive from stochastic timing of single-cell transcription.* Cell, 2009. **138**(3): p. 489-501.
9. Ma, L., et al., *A plausible model for the digital response of p53 to DNA damage.* Proc Natl Acad Sci U S A, 2005. **102**(40): p. 14266-71.
10. Pedraza, J.M. and J. Paulsson, *Effects of molecular memory and bursting on fluctuations in gene expression.* Science, 2008. **319**(5861): p. 339-43.
11. Ciliberto, A., B. Novak, and J.J. Tyson, *Steady states and oscillations in the p53/Mdm2 network.* Cell Cycle, 2005. **4**(3): p. 488-93.
12. Geva-Zatorsky, N., et al., *Oscillations and variability in the p53 system.* Molecular Systems Biology, 2006. **2**: p. 2006 0033.
13. Kubbutat, M.H., S.N. Jones, and K.H. Vousden, *Regulation of p53 stability by Mdm2.* Nature, 1997. **387**(6630): p. 299-303.
14. Liu, M., et al., *Increase in p53 protein half-life in mouse keratinocytes following UV-B irradiation.* Carcinogenesis, 1994. **15**(6): p. 1089-92.
15. Batchelor, E., et al., *Recurrent initiation: a mechanism for triggering p53 pulses in response to DNA damage.* Molecular Cell, 2008. **30**(3): p. 277-89.
16. Park, J.H., et al., *The GAS41-PP2Cbeta complex dephosphorylates p53 at serine 366 and regulates its stability.* The Journal of biological chemistry, 2011. **286**(13): p. 10911-7.
17. Maki, C.G. and P.M. Howley, *Ubiquitination of p53 and p21 is differentially affected by ionizing and UV radiation.* Molecular and Cellular Biology, 1997. **17**(1): p. 355-63.
18. Li, M., et al., *Deubiquitination of p53 by HAUSP is an important pathway for p53 stabilization.* Nature, 2002. **416**(6881): p. 648-53.

19. Barak, Y., et al., *Regulation of mdm2 expression by p53: alternative promoters produce transcripts with nonidentical translation potential.* Genes Dev, 1994. **8**(15): p. 1739-49.
20. Wang, Y.V., et al., *Quantitative analyses reveal the importance of regulated Hdmx degradation for p53 activation.* Proc Natl Acad Sci U S A, 2007. **104**(30): p. 12365-70.
21. Stommel, J.M. and G.M. Wahl, *Accelerated MDM2 auto-degradation induced by DNA-damage kinases is required for p53 activation.* EMBO J, 2004. **23**(7): p. 1547-56.
22. Brooks, C.L. and W. Gu, *p53 ubiquitination: Mdm2 and beyond.* Molecular Cell, 2006. **21**(3): p. 307-15.
23. Clore, G.M., et al., *High-resolution structure of the oligomerization domain of p53 by multidimensional NMR.* Science, 1994. **265**(5170): p. 386-91.
24. McLure, K.G. and P.W. Lee, *How p53 binds DNA as a tetramer.* EMBO J, 1998. **17**(12): p. 3342-50.
25. Schon, O., et al., *Molecular mechanism of the interaction between MDM2 and p53.* Journal of Molecular Biology, 2002. **323**(3): p. 491-501.
26. Lai, Z., et al., *Human mdm2 mediates multiple mono-ubiquitination of p53 by a mechanism requiring enzyme isomerization.* J Biol Chem, 2001. **276**(33): p. 31357-67.
27. McVean, M., et al., *Increase in wild-type p53 stability and transactivational activity by the chemopreventive agent apigenin in keratinocytes.* Carcinogenesis, 2000. **21**(4): p. 633-9.
28. Nicholls, C.D., et al., *Biogenesis of p53 involves cotranslational dimerization of monomers and posttranslational dimerization of dimers. Implications on the dominant negative effect.* J Biol Chem, 2002. **277**(15): p. 12937-45.
29. Weinberg, R.L., et al., *Regulation of DNA binding of p53 by its C-terminal domain.* Journal of Molecular Biology, 2004. **342**(3): p. 801-11.
30. Hsing, A., D.V. Faller, and C. Vaziri, *DNA-damaging aryl hydrocarbons induce Mdm2 expression via p53-independent post-transcriptional mechanisms.* The Journal of biological chemistry, 2000. **275**(34): p. 26024-31.
31. Cai, X. and Z.M. Yuan, *Stochastic modeling and simulation of the p53-MDM2/MDMX loop.* Journal of computational biology : a journal of computational molecular cell biology, 2009. **16**(7): p. 917-33.
